# Supplementary material for: TRIM2 E3 ligase substrate discovery reveals zinc-mediated regulation of TMEM106B in the endolysosomal pathway
Source: EMBO Rep. 2026 Jan 3;27(3):729–47. doi: 10.1038/s44319-025-00667-3 (PMC12894719; doi:10.1038/s44319-025-00667-3)
Supplement: Supplementary file 2 — Appendix [file 44319_2025_667_MOESM2_ESM.pdf]

Appendix

Zinc-mediated regulation of TMEM106B function in the endolysosomal pathway revealed through the systematic identification of TRIM2 ubiquitination substrates

Cecilia Perez-Borrajero[1,#], Frank Stein[2], Kristian Schweimer[3], Mandy Rettel[2], Jennifer J. Schwarz[2], Per Haberkant[2], Karine Lapouge[4], Jesse Gayk[1], Thomas Hoffmann[1], Sagar Bhogaraju[5], Mikhail Savitski[1,2], Julia Mahamid[1,6], and Janosch Hennig[1,3,#]

[1]Molecular Systems Biology Unit, European Molecular Biology Laboratory, Heidelberg, Germany

[2]Proteomics Core Facility, European Molecular Biology Laboratory, Heidelberg, Germany

[3]Chair of Biochemistry IV, Biophysical Chemistry, University of Bayreuth, Bayreuth, Germany

[4]Protein Expression and Purification Core Facility, European Molecular Biology Laboratory, Heidelberg, Germany

[5]Structural Biology Unit, European Molecular Biology Laboratory, Grenoble, France

[6]Cell Biology and Biophysics Unit, European Molecular Biology Laboratory, Heidelberg, Germany

[#]co-corresponding authors: cecilia.perez@embl.de, janosch.hennig@uni-bayreuth.de

Table of Content

Appendix Figure S1: Related to Figure 1; TRIM2 expression in HEK 293-T cells……………….…………..……..….3

Appendix Figure S2: Related to Figure 2; *in vitro* ubiquitination assays and co-transfections…….………..……...….5

Appendix Figure S3: Related to Figure 3; biophysical studies of the TRIM2/TMEM106B interaction…………....…..7

Appendix Figure S4: Related to Figure 5; TMEM106B expression in HEK 293-T cells……………………........….9

Appendix Table S1: Structure calculation statistics corresponding to TMEM106B$^{54-92}$…..……….…..……………...11

References…………………………………………………………………………………………………….……..…12

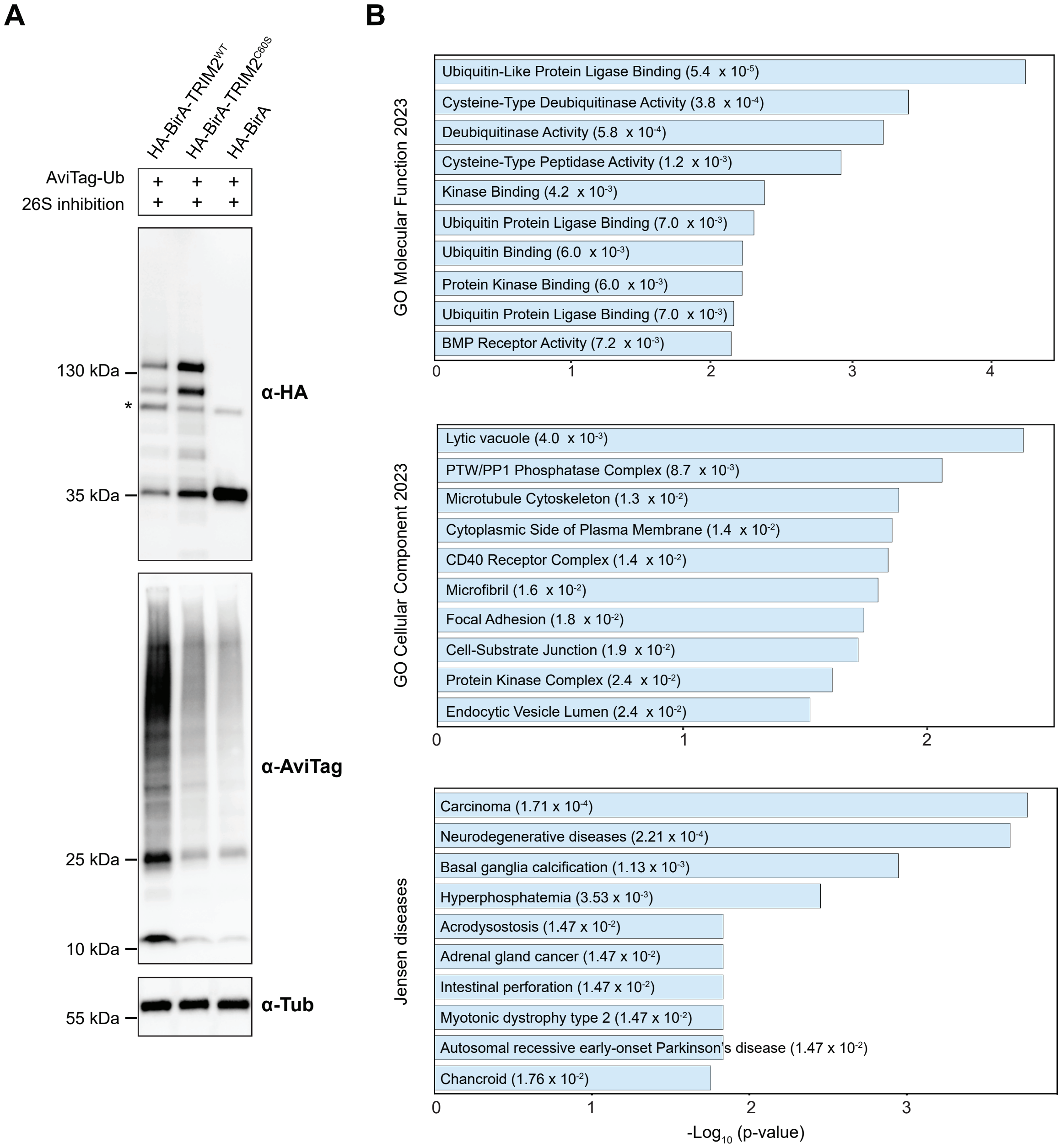

A
HA-BirA-TRIM2WT
HA-BirA-TRIM2C60S
HA-BirA
AviTag-Ub + + +
26S inhibition + + +
130 kDa
*
35 kDa
α-HA
α-AviTag
25 kDa
10 kDa
α-Tub
55 kDa
B
GO Molecular Function 2023
Ubiquitin-Like Protein Ligase Binding (5.4 x 10-5)
Cysteine-Type Deubiquitinase Activity (3.8 x 10-4)
Deubiquitinase Activity (5.8 x 10-4)
Cysteine-Type Peptidase Activity (1.2 x 10-3)
Kinase Binding (4.2 x 10-3)
Ubiquitin Protein Ligase Binding (7.0 x 10-3)
Ubiquitin Binding (6.0 x 10-3)
Protein Kinase Binding (6.0 x 10-3)
Ubiquitin Protein Ligase Binding (7.0 x 10-3)
BMP Receptor Activity (7.2 x 10-3)
GO Cellular Component 2023
Lytic vacuole (4.0 x 10-3)
PTW/PP1 Phosphatase Complex (8.7 x 10-3)
Microtubule Cytoskeleton (1.3 x 10-2)
Cytoplasmic Side of Plasma Membrane (1.4 x 10-2)
CD40 Receptor Complex (1.4 x 10-2)
Microfibril (1.6 x 10-2)
Focal Adhesion (1.8 x 10-2)
Cell-Substrate Junction (1.9 x 10-2)
Protein Kinase Complex (2.4 x 10-2)
Endocytic Vesicle Lumen (2.4 x 10-2)
Jensen diseases
Carcinoma (1.71 x 10-4)
Neurodegenerative diseases (2.21 x 10-4)
Basal ganglia calcification (1.13 x 10-3)
Hyperphosphatemia (3.53 x 10-3)
Acrodysostosis (1.47 x 10-2)
Adrenal gland cancer (1.47 x 10-2)
Intestinal perforation (1.47 x 10-2)
Myotonic dystrophy type 2 (1.47 x 10-2)
Autosomal recessive early-onset Parkinson's disease (1.47 x 10-2)
Chancroid (1.76 x 10-2)
-Log10 (p-value)

Appendix Figure S1: (A) Cotransfection of AviTag-Ub and HA-BirA plasmids in HEK 293-T cells. The expression levels of HA-BirA-containing gene constructs (above) and AviTag-Ub (below) upon transfection of HEK 293-T cells were assessed using immunoblotting. Equal amounts of protein were loaded and tested in parallel SDS-PAGE runs, with tubulin serving as a loading control. Proteasome inhibition was important to improve TRIM2 expression levels. TRIM2 shows multiple protein bands, including possible natural proteoforms and a product consistent with HA-BirA. The asterisk corresponds to non-specific binding of the anti-HA antibody. **(B)** The identified TRIM2 ubiquitination substrates (n=29) were used to analyze enrichment of cellular functions, compartments, and diseases using the Enrichr server (Kuleshov et al., 2016). The p-values reported were determined using Fisher's hypergeometric test.

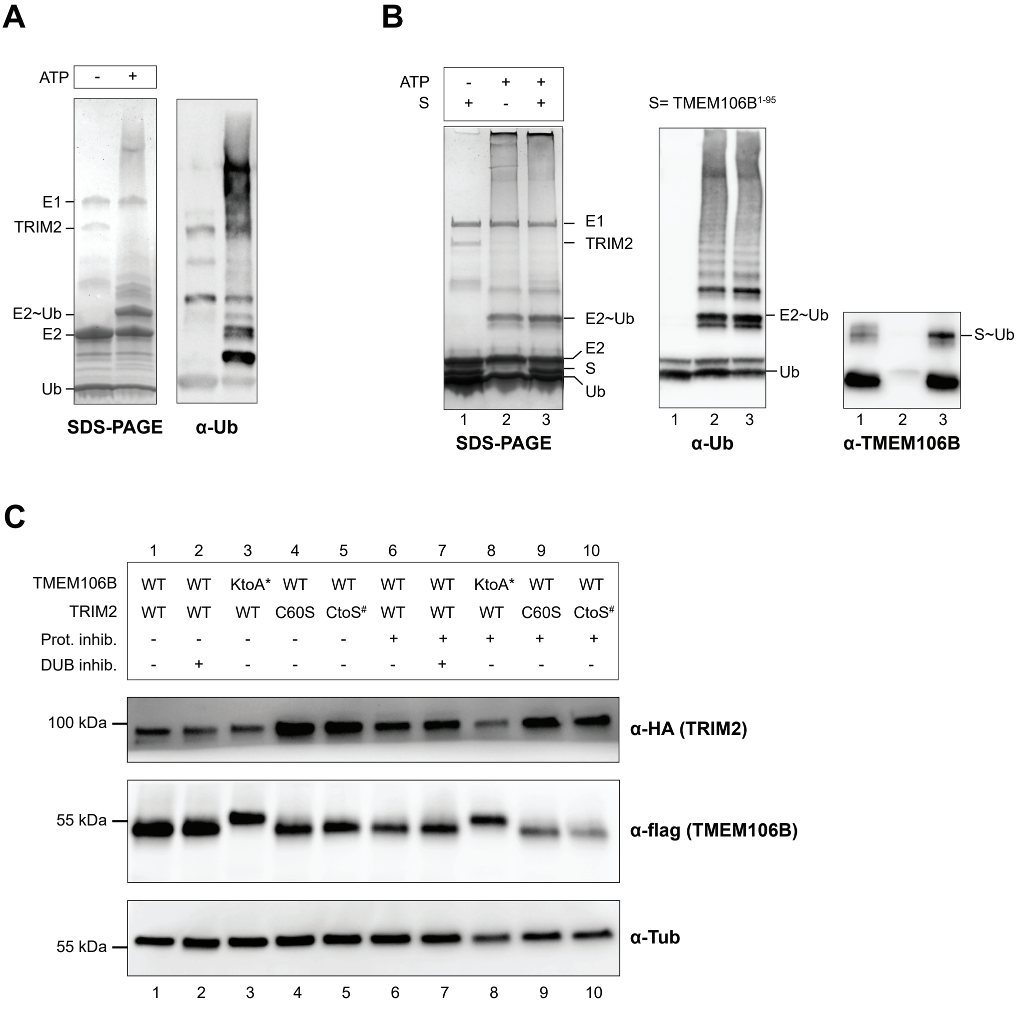

*KtoA refers to two point mutations: K3A/K14A

#CtoS refers to two point mutations: C23S/C60S

Appendix Figure S2: (A, B) *In vitro* autoubiquitination assays using human proteins obtained from *E. coli* recombinant expression. The extent of the reaction upon addition of 5 mM ATP-$MgCl_2$ was assessed by Coomassie stained SDS-PAGE or immunoblotting against ubiquitin and TMEM106B. **(C)** HA-tagged TRIM2 and flag-tagged TMEM106B variants were co-expressed in HEK 293-T cells using transient transfections, and the protein levels assessed with immunoblotting after cell lysis. Proteasome (Carfilzomib, 0.5 μM) and deubiquitinase inhibition (DUB, PR-619, Calbiochem, 25 μM) was performed where indicated, for ~ 3 hours, 21 hours post transfection. Note that TMEM106B$^{K3A/K14A}$ runs at a slightly higher MW relative to TMEM106B^{WT}.

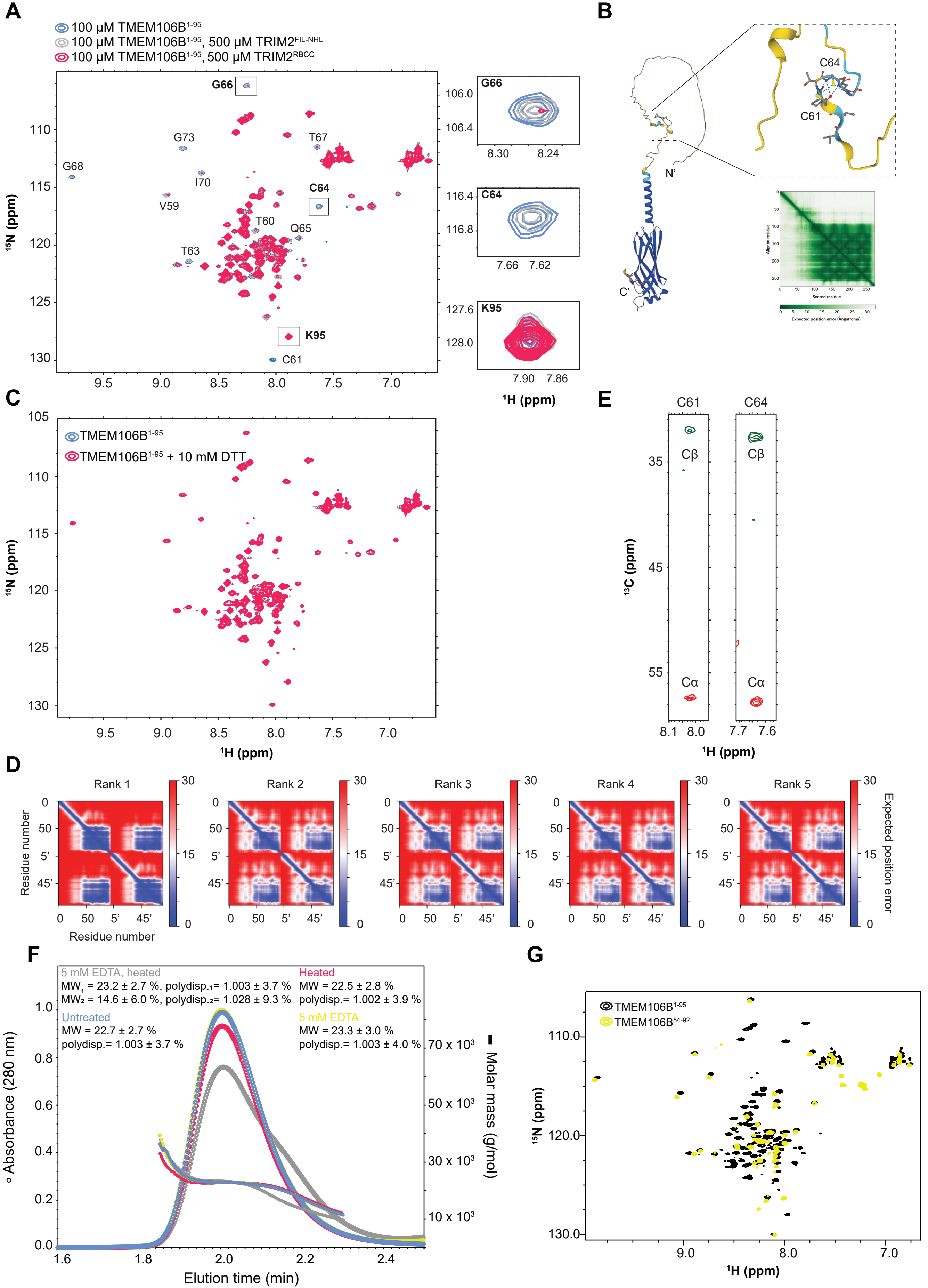

A
100 µM TMEM106B1-95
100 µM TMEM106B1-95, 500 µM TRIM2FIL-NHL
100 µM TMEM106B1-95, 500 µM TRIM2RBCC
G66
T67
G73
G68
I70
C64
V59
T60
Q65
T63
K95
C61
15N (ppm)
1H (ppm)
B
C64
C61
N'
C'
Expected position error (Ångströms)
C
TMEM106B1-95
TMEM106B1-95 + 10 mM DTT
E
C61
C64
Cβ
Cα
13C (ppm)
D
Rank 1
Rank 2
Rank 3
Rank 4
Rank 5
Residue number
Expected position error
F
5 mM EDTA, heated
MW1 = 23.2 ± 2.7 %, polydisp.1= 1.003 ± 3.7 %
MW2 = 14.6 ± 6.0 %, polydisp.2= 1.028 ± 9.3 %
Heated
MW = 22.5 ± 2.8 %
polydisp.= 1.002 ± 3.9 %
Untreated
MW = 22.7 ± 2.7 %
polydisp.= 1.003 ± 3.7 %
5 mM EDTA
MW = 23.3 ± 3.0 %
polydisp.= 1.003 ± 4.0 %
Absorbance (280 nm)
Molar mass (g/mol)
Elution time (min)
G
TMEM106B1-95
TMEM106B54-92

Appendix Figure S3: (A) TRIM2 binds TMEM106B$^{1-95}$ directly. The ^{1}H-^{15}N HSQC of ^{15}N-labelled TMEM106B$^{1-95}$ was recorded in the absence (blue) and presence of 5-fold molar excess FIL-NHL domains (grey), or the RBCC region (red), and the resulting spectra are shown overlaid. Amide peaks exhibiting the clearest changes are indicated and correspond to a region centered around residue 60 (see also **Fig. 3b** for quantification). Three peaks exhibiting line broadening to varying degrees are highlighted in zoomed-in views (right). **(B)** AlphaFold prediction of monomeric human TMEM106B as found in the Protein Structure Database show low position errors (plotted in green) of the luminal and transmembrane regions, while the prediction of the disordered N-terminal domain suggests that cysteine residues 61 and 64 may form a disulfide bond (zoomed in the inset). **(C)** Addition of reducing agent did not influence the conformational state of TMEM106B$^{1-95}$. Shown are overlaid ^{1}H-^{15}N HSQC spectra in the absence (blue) and presence (red) of 10 mM DTT. **(D)** Plots of expected position errors of predicted homodimeric TMEM106B$^{1-95}$ using AlphaFold-Multimer, corresponding to the top 5 ranked models(Evans et al., 2022). The C-terminal region (residues ~50-95) is consistently predicted with lower errors and corresponds to the dimeric interface. **(E)** The chemical shifts of ^{13}C$^{\beta}$ cysteine residues 61 and 64 were obtained from standard three-dimensional backbone experiments and have a value of ~ 32 ppm, consistent with their involvement in zinc coordination. Shown are strip plots extracted from 3D experiments detecting ^{13}C$^{\beta}$ and ^{13}C$^{\alpha}$ atoms. **(F)** SEC-MALS experiments using TMEM106B$^{1-95}$ samples treated with heat (2 hours, 45 ºC), in the presence and absence of 5 mM EDTA. The samples were injected at a concentration of ~ 1 mg/mL in all cases. The presence of EDTA promoted the formation of a smaller MW species upon heating the sample, consistent with the role of a metal ion in mediating dimerization. **(G)** A peptide corresponding to TMEM106B$^{54-92}$ supplemented with zinc exhibits the characteristic chemical shifts found in the longer TMEM106B$^{1-95}$ region. Shown are overlaid ^{1}H-^{15}N-HSQC spectra of the shorter (yellow) and longer (black) polypeptides.

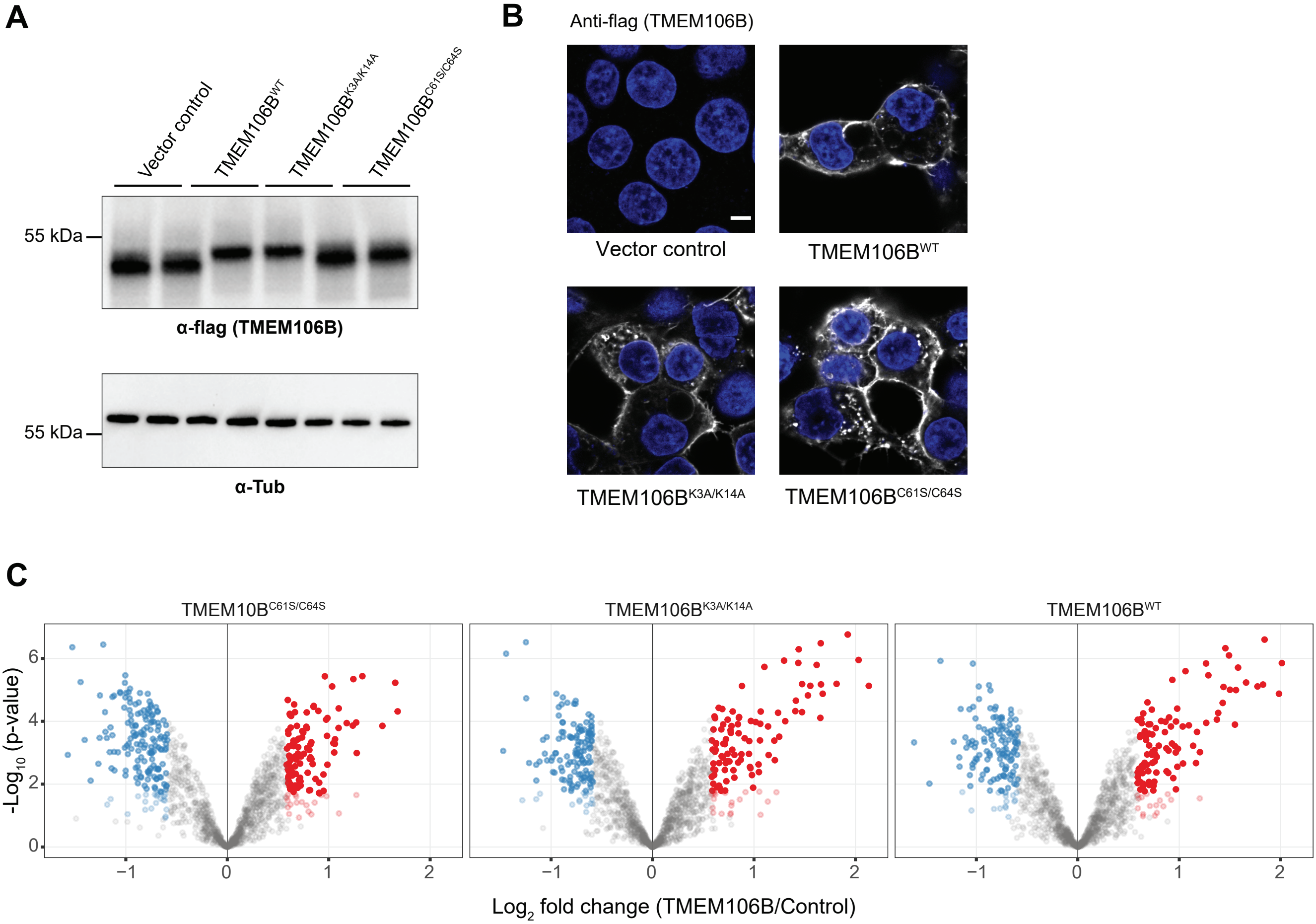
A
Vector control
TMEM106B^WT
TMEM106B^K3A/K14A
TMEM106B^C61S/C64S
55 kDa
α-flag (TMEM106B)
55 kDa
α-Tub
B
Anti-flag (TMEM106B)
Vector control
TMEM106B^WT
TMEM106B^K3A/K14A
TMEM106B^C61S/C64S
C
TMEM10B^C61S/C64S
TMEM106B^K3A/K14A
TMEM106B^WT
-Log10 (p-value)
Log2 fold change (TMEM106B/Control)

Appendix Figure S4: Variants of TMEM106B do not show significant changes in expression levels or localization upon overexpression. **(A)** HEK 293-T cells were transfected with flag-tagged TMEM106B for 48 h, and the expression levels assessed with immunoblotting against the flag-tag (above), or tubulin serving as a loading control (below). Two biological replicates for each condition were performed and loaded on separate lanes of an SDS-PAGE gel. **(B)** Representative images of HEK 293-T cells transfected with TMEM106B variants following fixation and immunostaining against the flag-tag (white). The blue color marks the nucleus of each cell which was counterstained with DAPI. The same scale bar was used in all images and corresponds to 5 μm. **(C)** Volcano scatter plots of proteins identified using MS following co-immunoprecipitation of TMEM106B variants expressed in HEK 293-T cells, relative to negative control (related to Figure 5). Proteins colored in red represent putative TMEM106B interactors, while those in blue were enriched in the control. The p-values were calculated using limma's empirical Bayes-moderated t-test (n=3).

Appendix Table S1: Structure calculation statistics corresponding to TMEM106B$^{54\text{-}92}$ derived from NMR spectroscopy (PDB: 9GI8)

**Conformational Restraints**	
NOE distance restraints	
total	834
short range |i-j| <= 1	532
medium-range, |i-j| < 5	86
long range, |i-j| >= 5	216
ϕ/ψ dihedral restraints from TALOS	14/14
Hydrogen bond restraints (upper/lower)	8/8
**Structure statistics**	
CYANA target function (Å^2)	1.3
Number NOE violations > 0.1 Å	6
Maximum NOE violation (Å)	0.14
Dihedral angle violations > 2.5°	0
Ramachandran plot statistics	
Most favored /additional allowed/ generously allowed/disallowed regions (%)	57.1 / 42.8 / 0.0 / 0.2
Coordinate precision (RMSD to mean coordinates)	
Backbone (Asp^{57A}-Pro^{71A} / Asp^{57B}-Pro^{71B}) (Å)	0.1
Heavy atoms	0.43

References:

Evans, R., O'Neill, M., Pritzel, A., Antropova, N., Senior, A., Green, T., Žídek, A., Bates, R., Blackwell, S., Yim, J., Ronneberger, O., Bodenstein, S., Zielinski, M., Bridgland, A., Potapenko, A., Cowie, A., Tunyasuvunakool, K., Jain, R., Clancy, E., … Hassabis, D. (2022). Protein complex prediction with AlphaFold-Multimer. *BioRxiv*, 2021.10.04.463034. https://doi.org/10.1101/2021.10.04.463034

Kuleshov, M. V., Jones, M. R., Rouillard, A. D., Fernandez, N. F., Duan, Q., Wang, Z., Koplev, S., Jenkins, S. L., Jagodnik, K. M., Lachmann, A., McDermott, M. G., Monteiro, C. D., Gundersen, G. W., & Maayan, A. (2016). Enrichr: a comprehensive gene set enrichment analysis web server 2016 update. *Nucleic Acids Research*, *44*(1), W90–W97. https://doi.org/10.1093/NAR/GKW377
