# Supplementary material for: TRIM2 E3 ligase substrate discovery reveals zinc-mediated regulation of TMEM106B in the endolysosomal pathway
Source: EMBO Rep. 2026 Jan 3;27(3):729–47. doi: 10.1038/s44319-025-00667-3 (PMC12894719; doi:10.1038/s44319-025-00667-3)
Supplement: Supplementary file 7 — Source data Fig. 1 [file 44319_2025_667_MOESM7_ESM.zip › Source_Data_Figure1/README.rtf]

Figure 1A: Schematic diagram, source data not applicable.Figure 1B: Western blot, source data provided. The region used in the manuscript has been highlighted in red. Figure 1C: Volcano scatter plot from quantitative MS data deposited in a repository (see data availability section).Figure 1D: Diagram from analysis of quantitative MS data, see above (1C) for source data. 
