# Supplementary figures and images for: TRIM2 E3 ligase substrate discovery reveals zinc-mediated regulation of TMEM106B in the endolysosomal pathway
Source: EMBO Rep. 2026 Jan 3;27(3):729–47. doi: 10.1038/s44319-025-00667-3 (PMC12894719; doi:10.1038/s44319-025-00667-3)

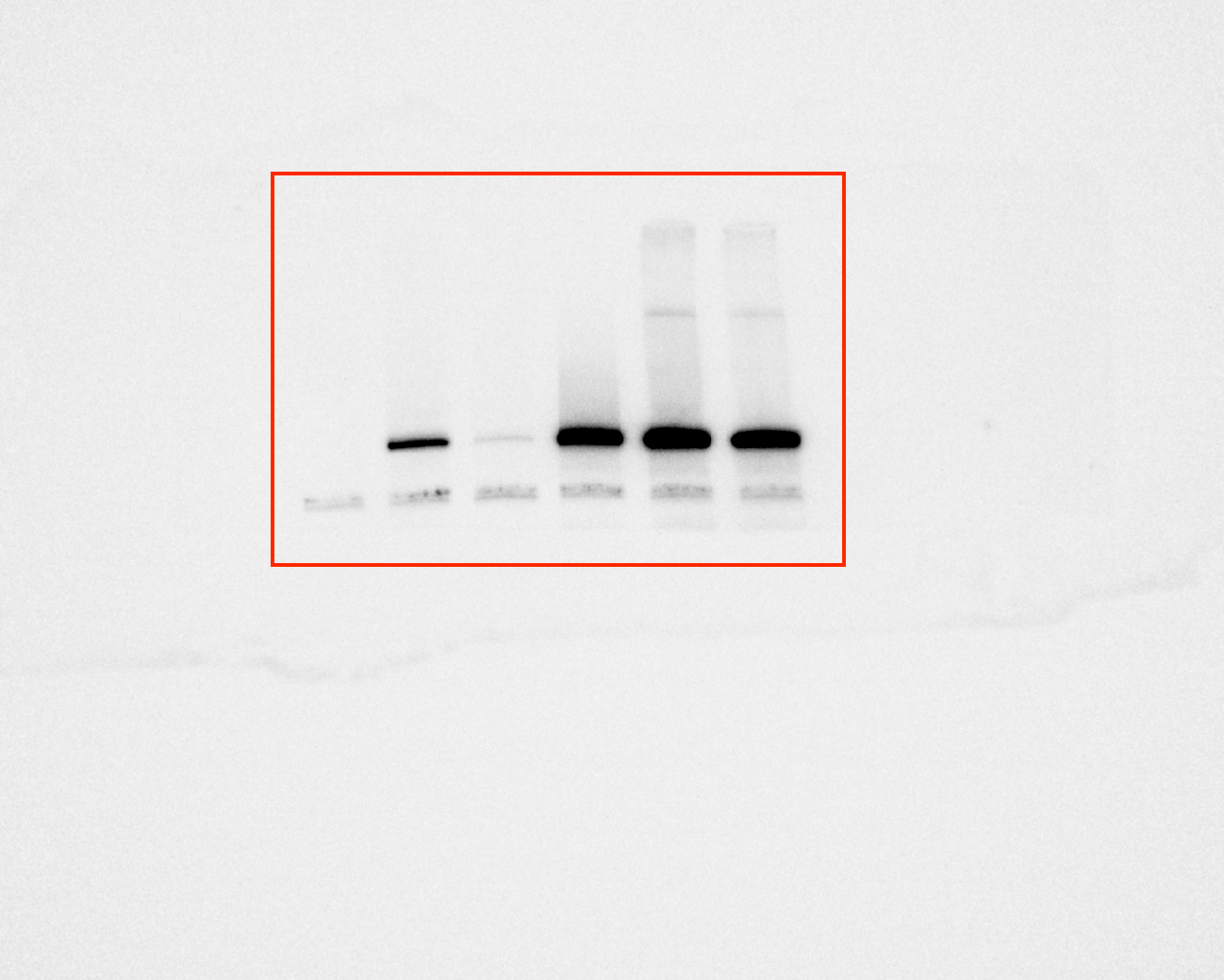

Supplement: Supplementary file 7 — Source data Fig. 1 [file 44319_2025_667_MOESM7_ESM.zip › Source_Data_Figure1/1B/WB_TRIM2.tiff]

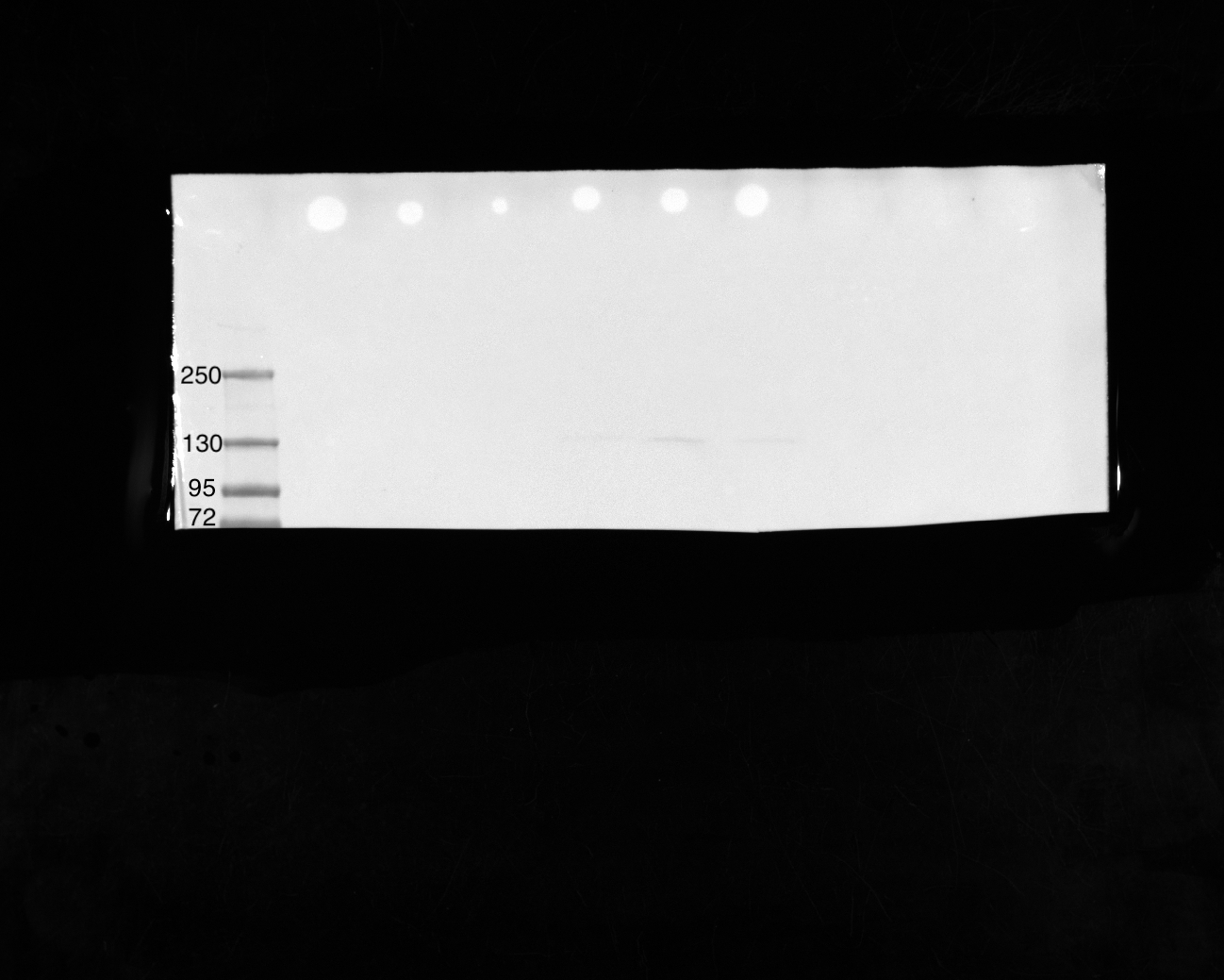

Supplement: Supplementary file 7 — Source data Fig. 1 [file 44319_2025_667_MOESM7_ESM.zip › Source_Data_Figure1/1B/Ladder_TRIM2.tiff]

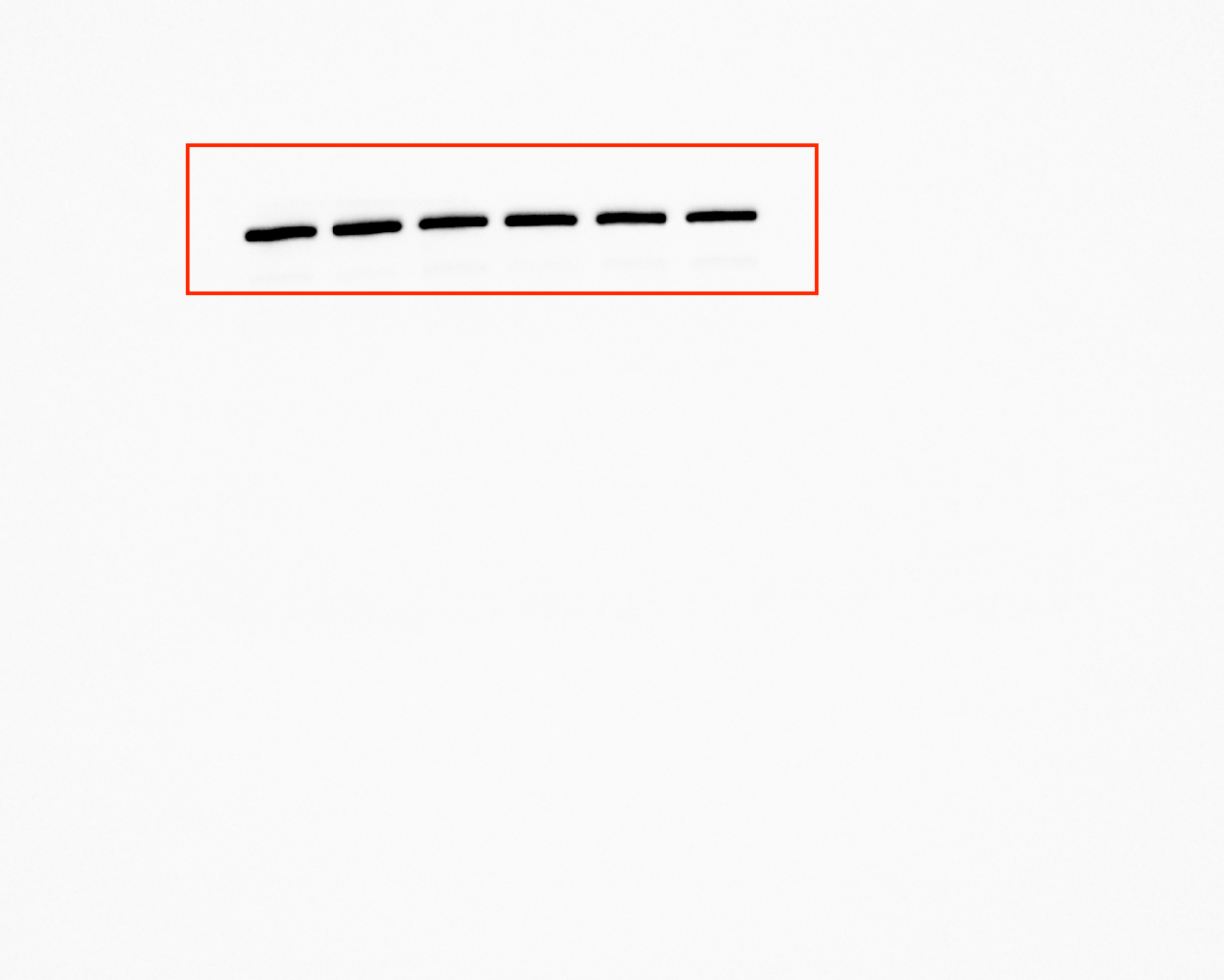

Supplement: Supplementary file 7 — Source data Fig. 1 [file 44319_2025_667_MOESM7_ESM.zip › Source_Data_Figure1/1B/WB_Tubulin.tiff]

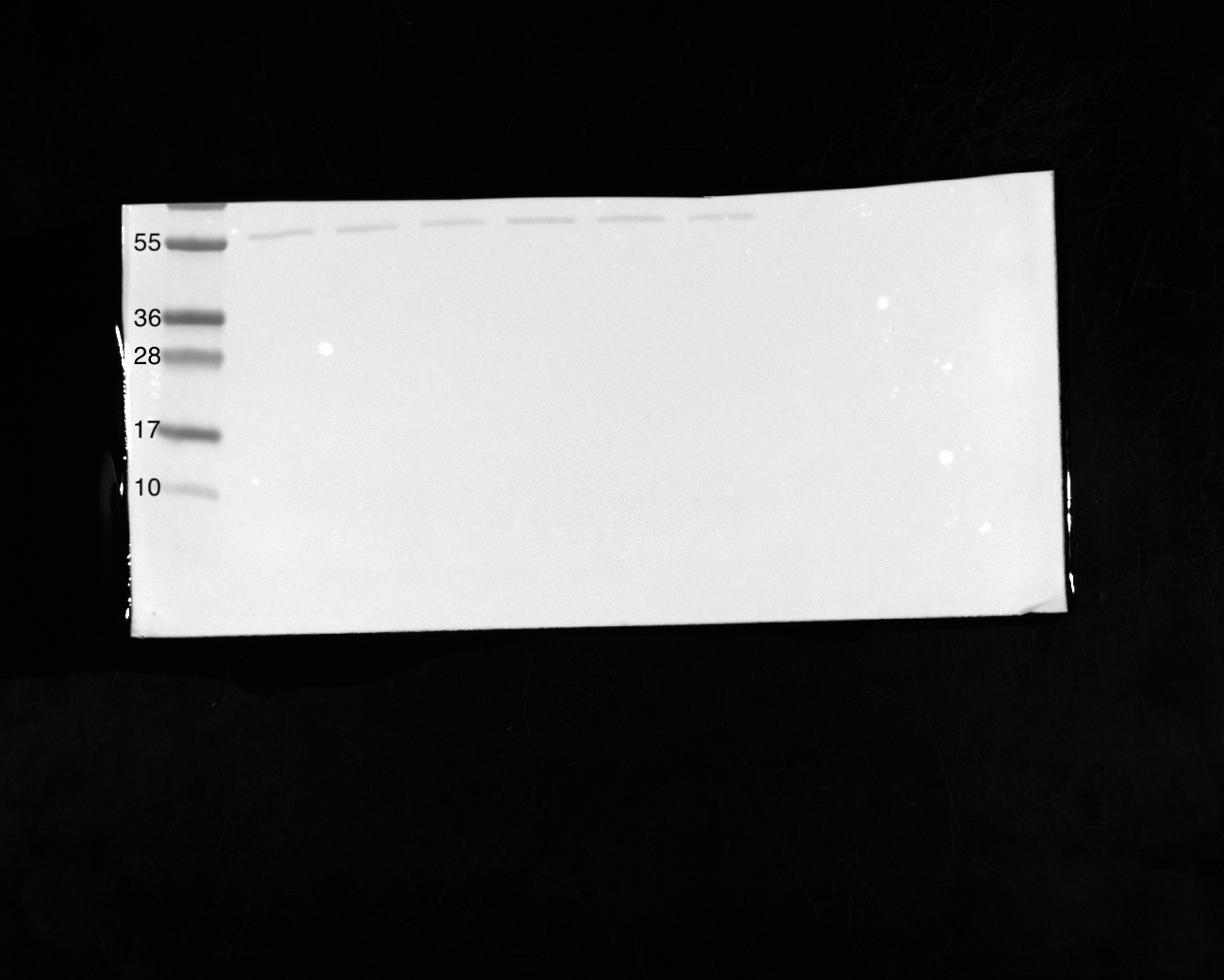

Supplement: Supplementary file 7 — Source data Fig. 1 [file 44319_2025_667_MOESM7_ESM.zip › Source_Data_Figure1/1B/Ladder_Tubulin.tiff]

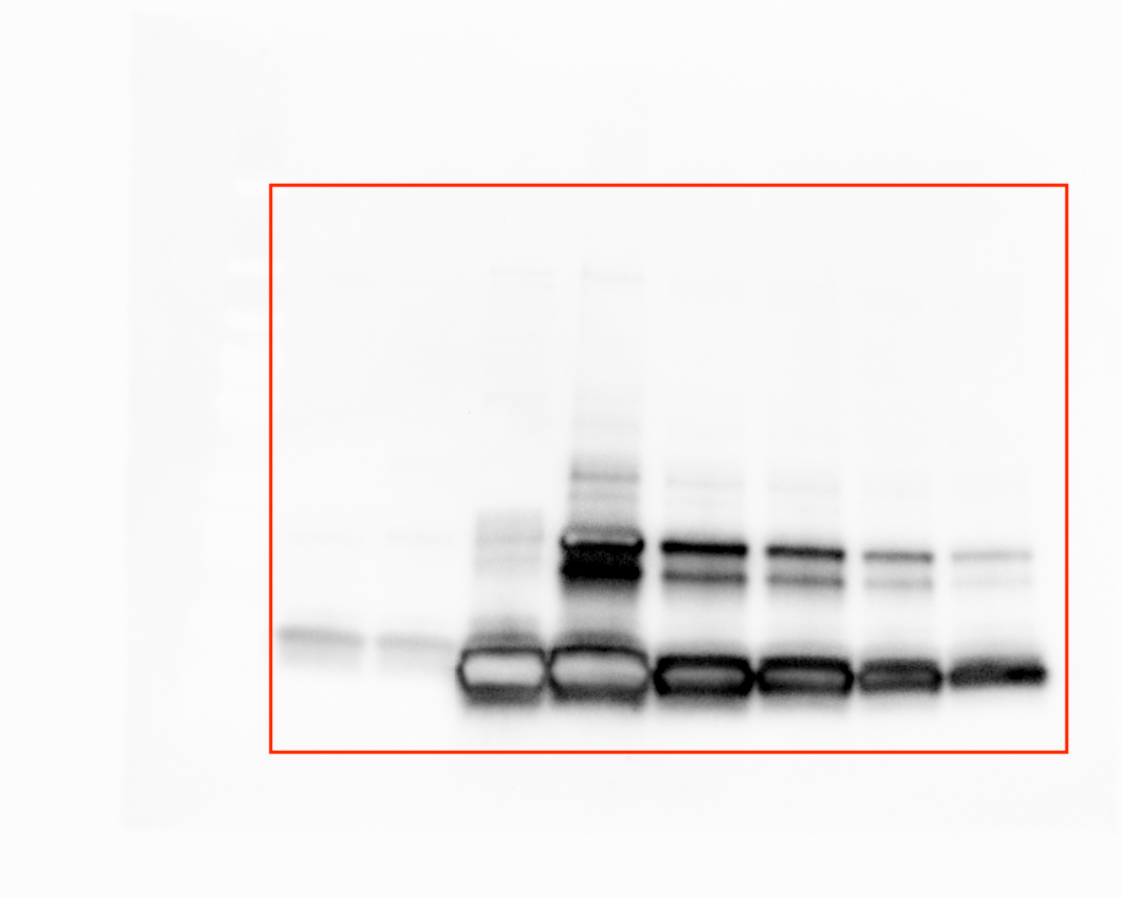

Supplement: Supplementary file 8 — Source data Fig. 2 [file 44319_2025_667_MOESM8_ESM.zip › Source_Data_Figure2/2C/TMEM106B_Ub_concentration.tif]

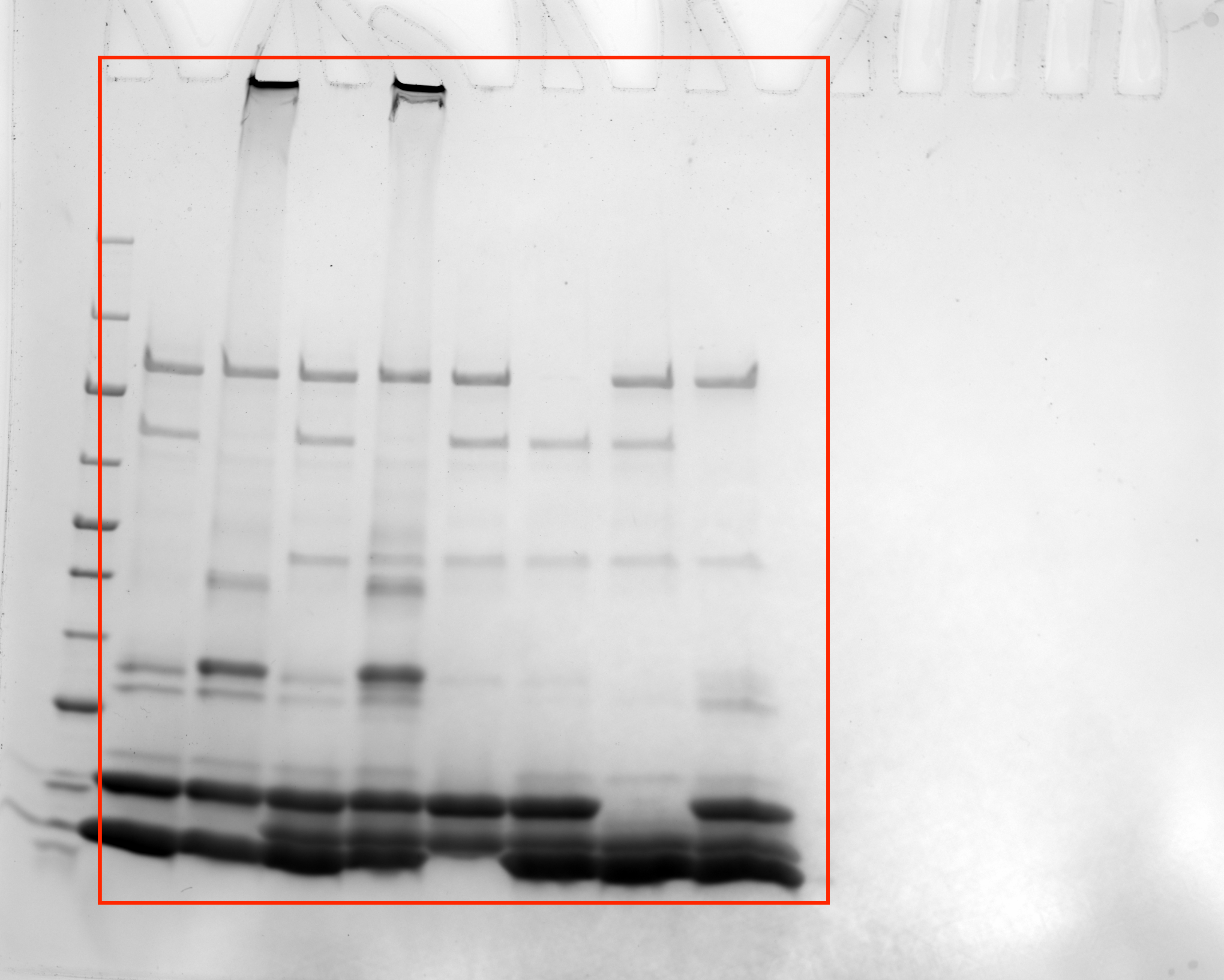

Supplement: Supplementary file 8 — Source data Fig. 2 [file 44319_2025_667_MOESM8_ESM.zip › Source_Data_Figure2/2B/SDS-PAGE_invitro.tif]

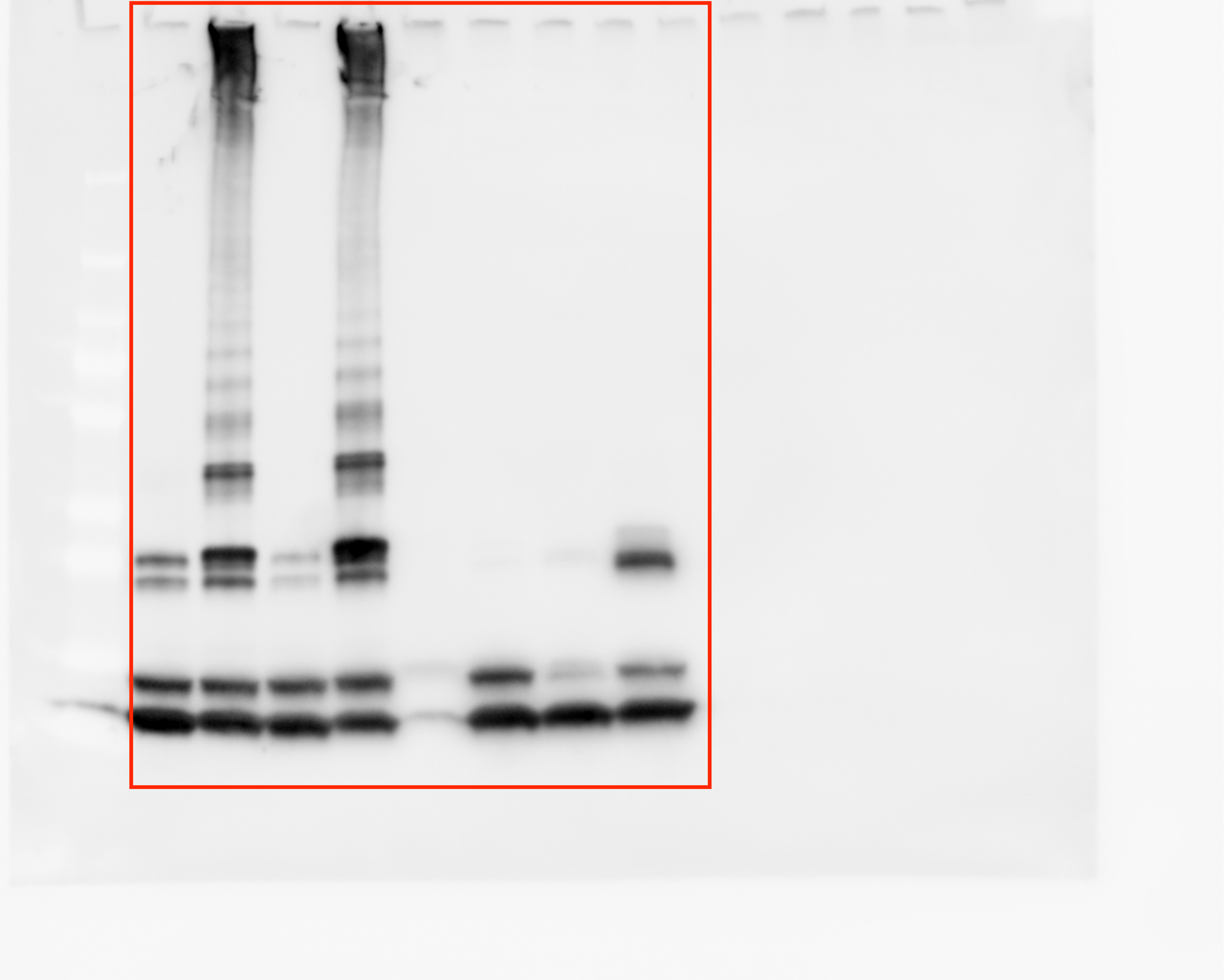

Supplement: Supplementary file 8 — Source data Fig. 2 [file 44319_2025_667_MOESM8_ESM.zip › Source_Data_Figure2/2B/Ub_WB_Invitro_assay.tif]

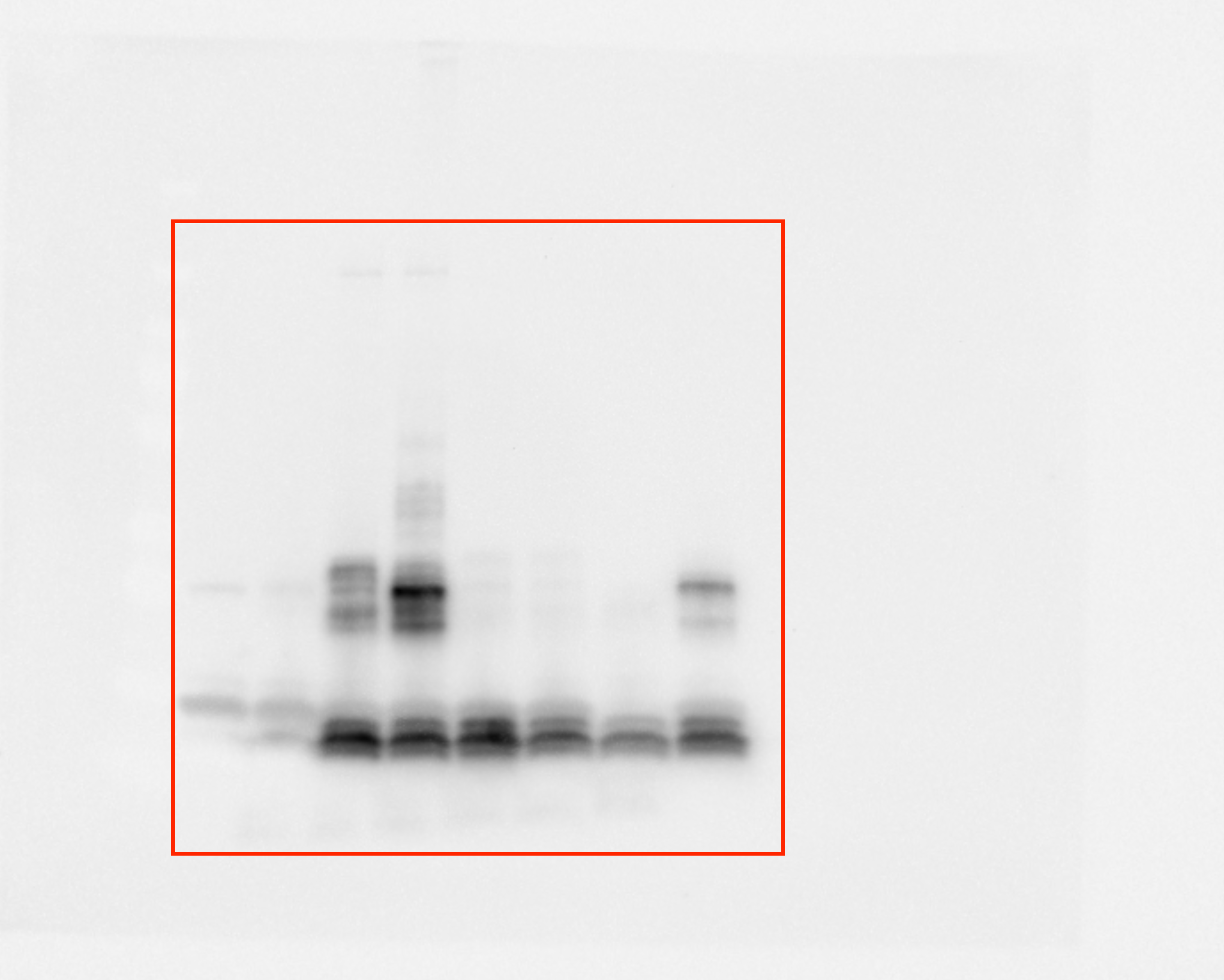

Supplement: Supplementary file 8 — Source data Fig. 2 [file 44319_2025_667_MOESM8_ESM.zip › Source_Data_Figure2/2B/TMEM106B_WB_Invitro_assay.tif]

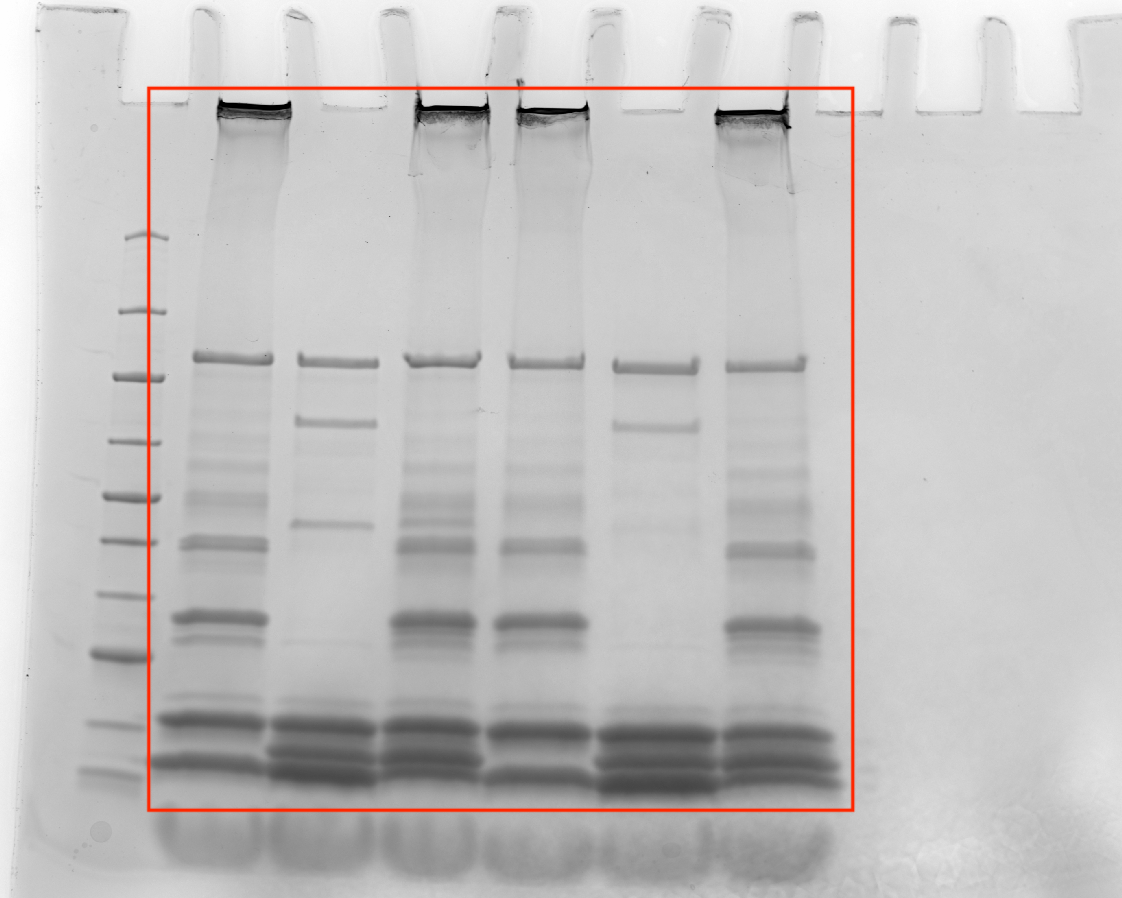

Supplement: Supplementary file 10 — Source data Fig. 4 [file 44319_2025_667_MOESM10_ESM.zip › Source_Data_Figure4/4C/TMEM_comparison_WT_mono_SDS-PAGE.tif]

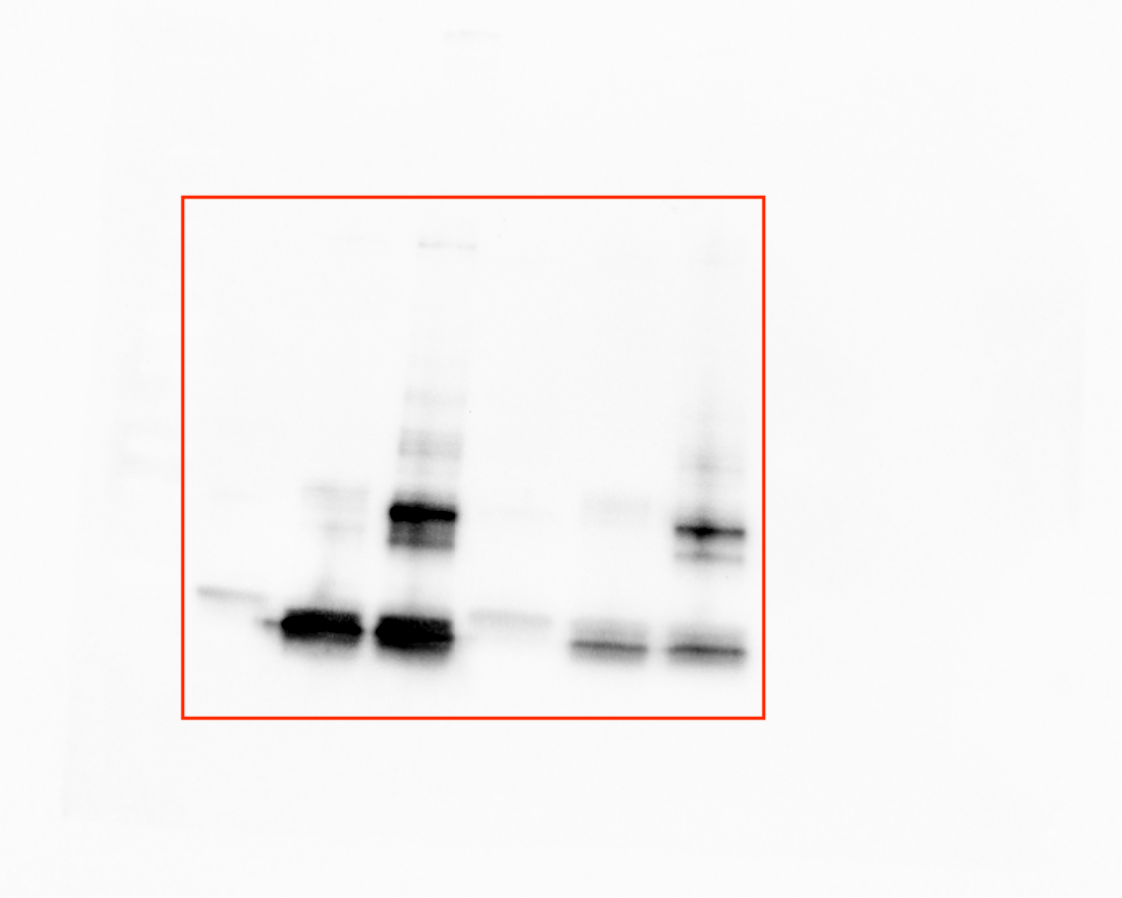

Supplement: Supplementary file 10 — Source data Fig. 4 [file 44319_2025_667_MOESM10_ESM.zip › Source_Data_Figure4/4C/Comparison_WT_Monomeric_TMEM_Ub.tif]

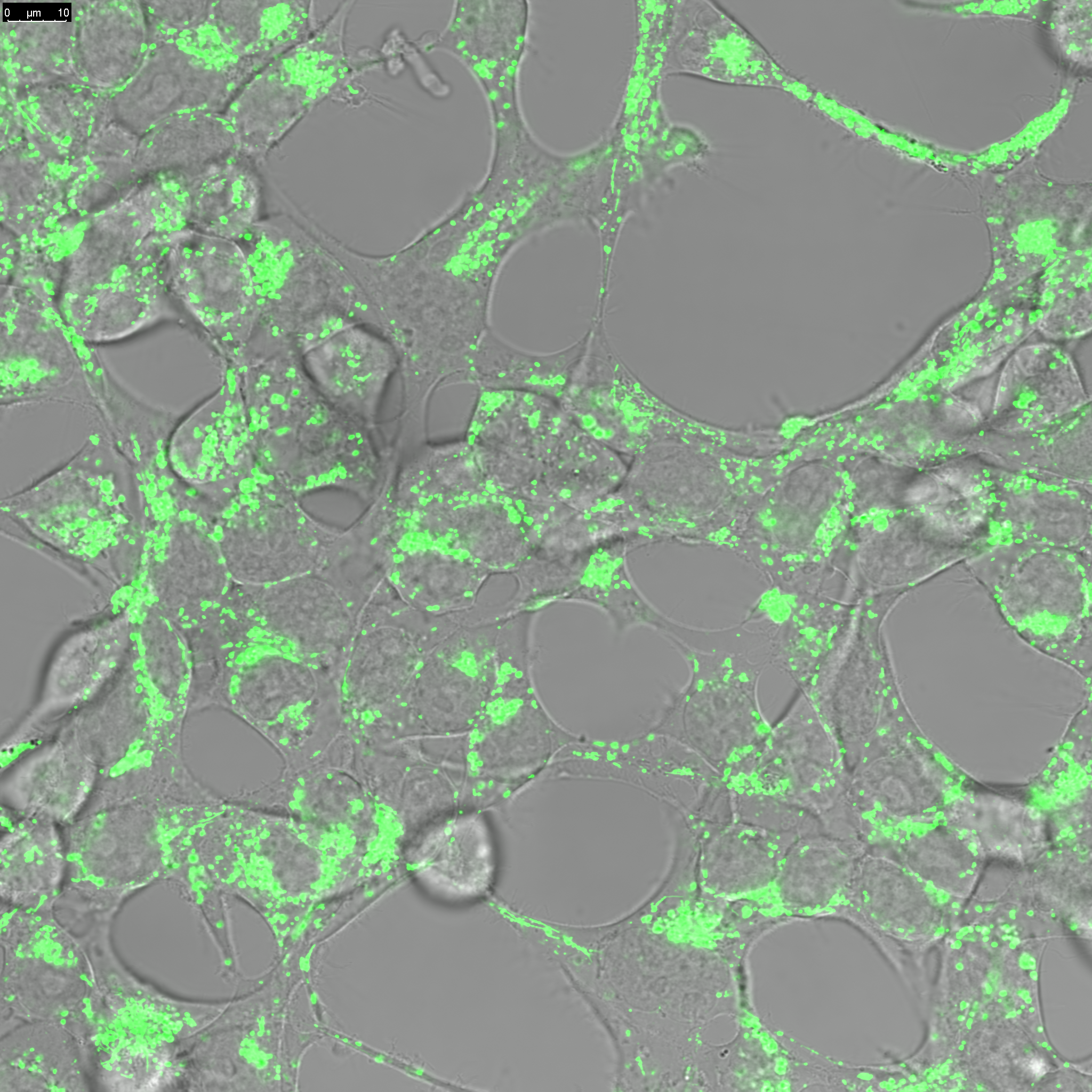

Supplement: Supplementary file 11 — Source data Fig. 5 [file 44319_2025_667_MOESM11_ESM.zip › Source_Data_Figure5/5A/Live_lysotrack_pCDNA_01.tif]

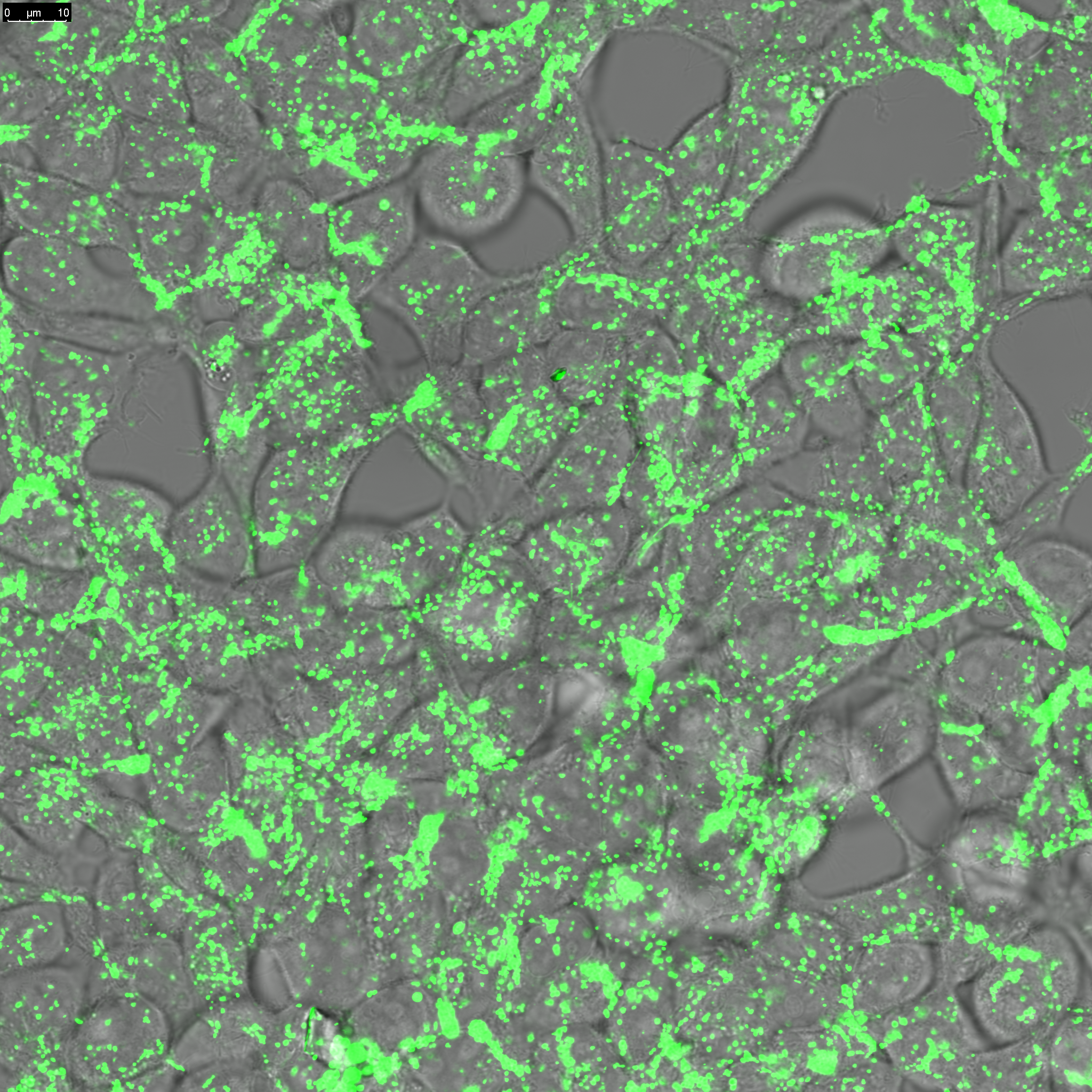

Supplement: Supplementary file 11 — Source data Fig. 5 [file 44319_2025_667_MOESM11_ESM.zip › Source_Data_Figure5/5A/Live_lysotrack_CtoS_03.tif]

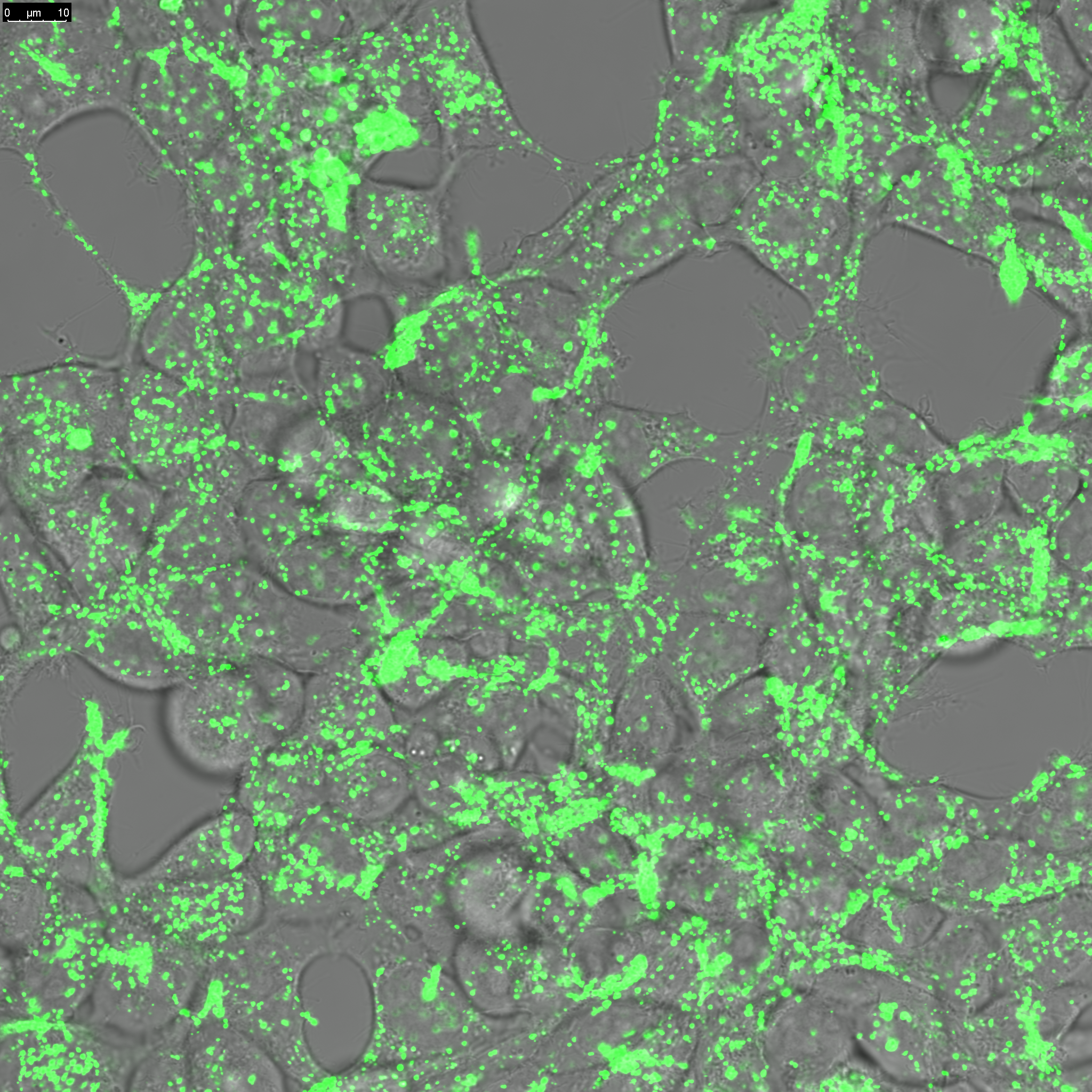

Supplement: Supplementary file 11 — Source data Fig. 5 [file 44319_2025_667_MOESM11_ESM.zip › Source_Data_Figure5/5A/Live_lysotrack_CtoS_02.tif]

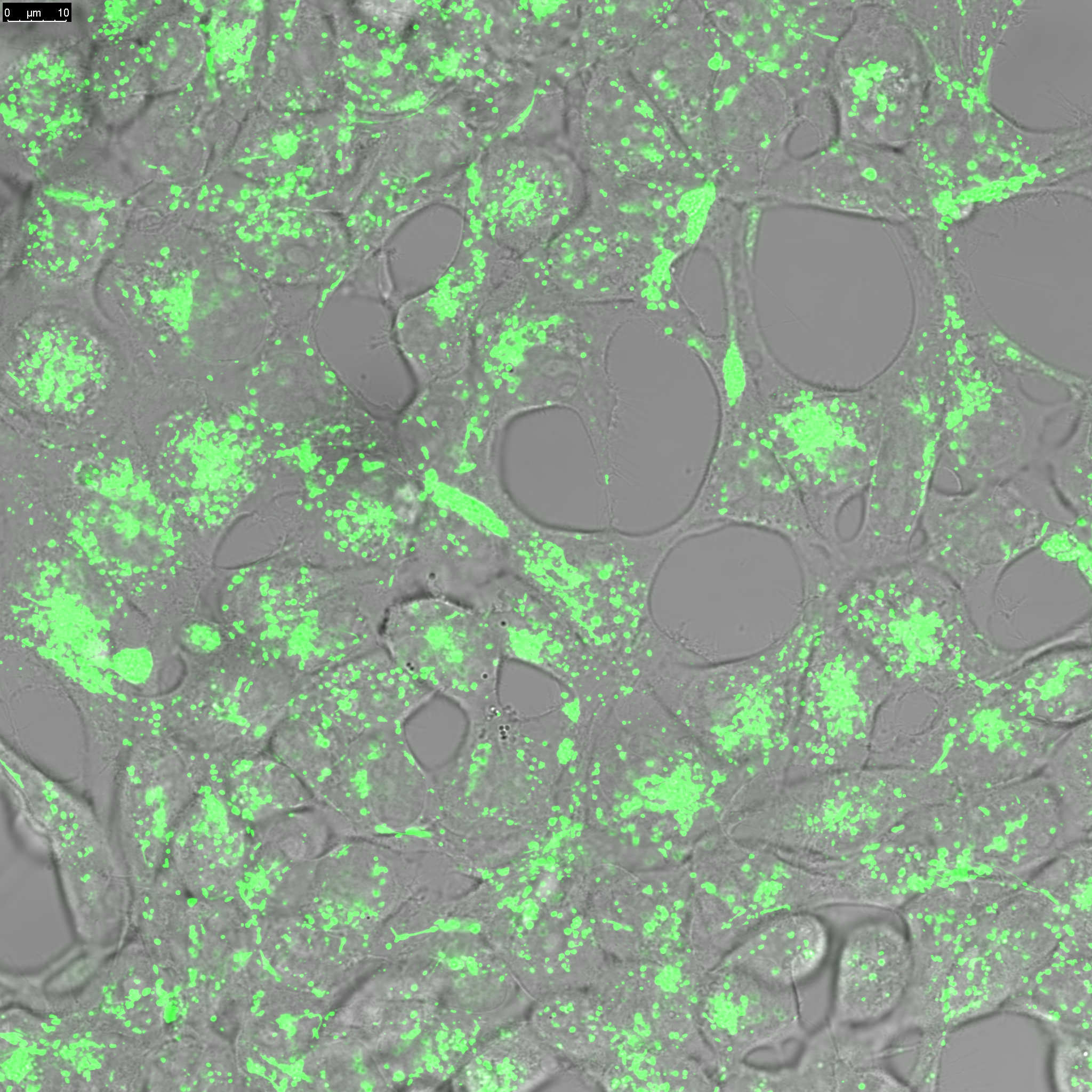

Supplement: Supplementary file 11 — Source data Fig. 5 [file 44319_2025_667_MOESM11_ESM.zip › Source_Data_Figure5/5A/Live_lysotrack_pCDNA_02.tif]

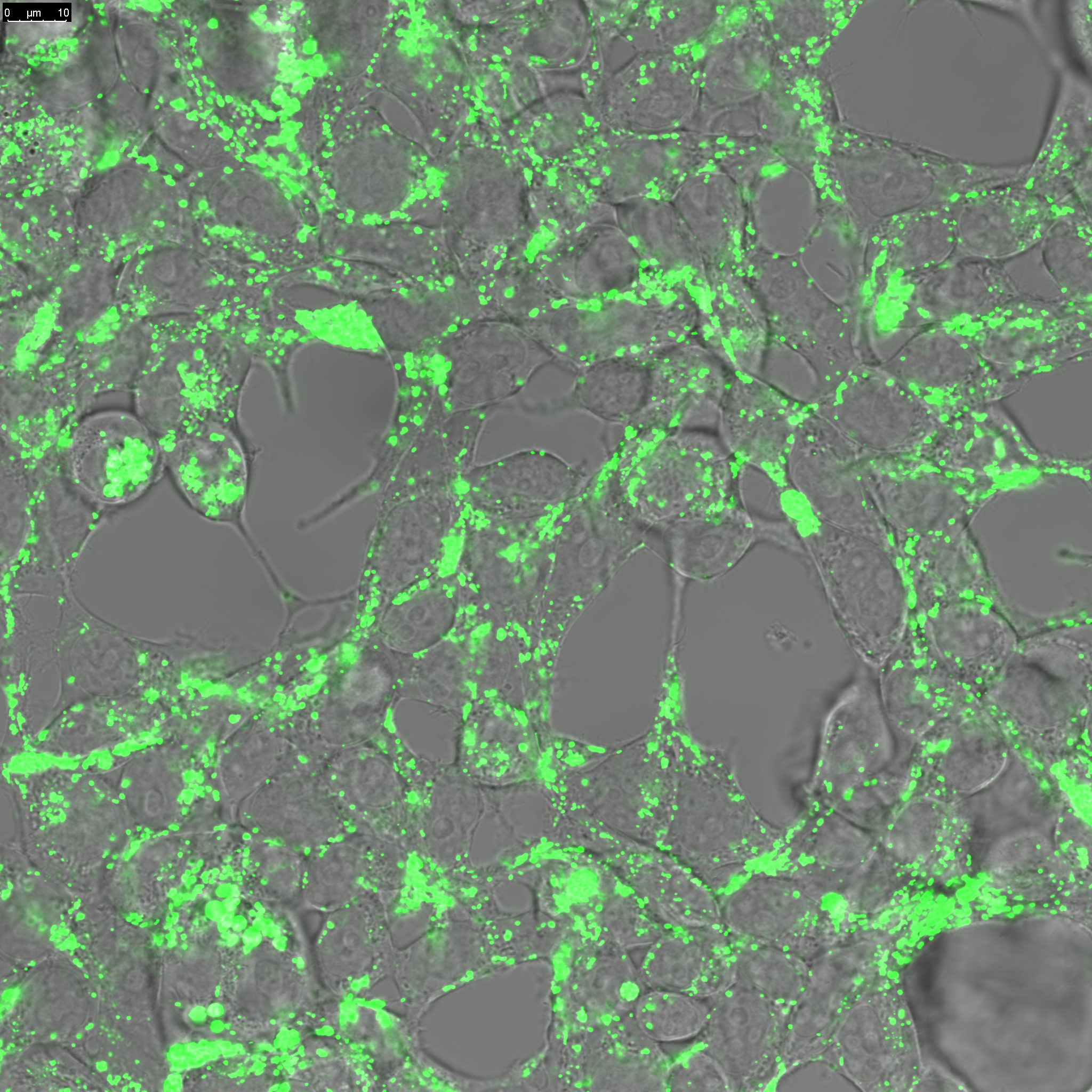

Supplement: Supplementary file 11 — Source data Fig. 5 [file 44319_2025_667_MOESM11_ESM.zip › Source_Data_Figure5/5A/Live_lysotrack_CtoS_01.tif]

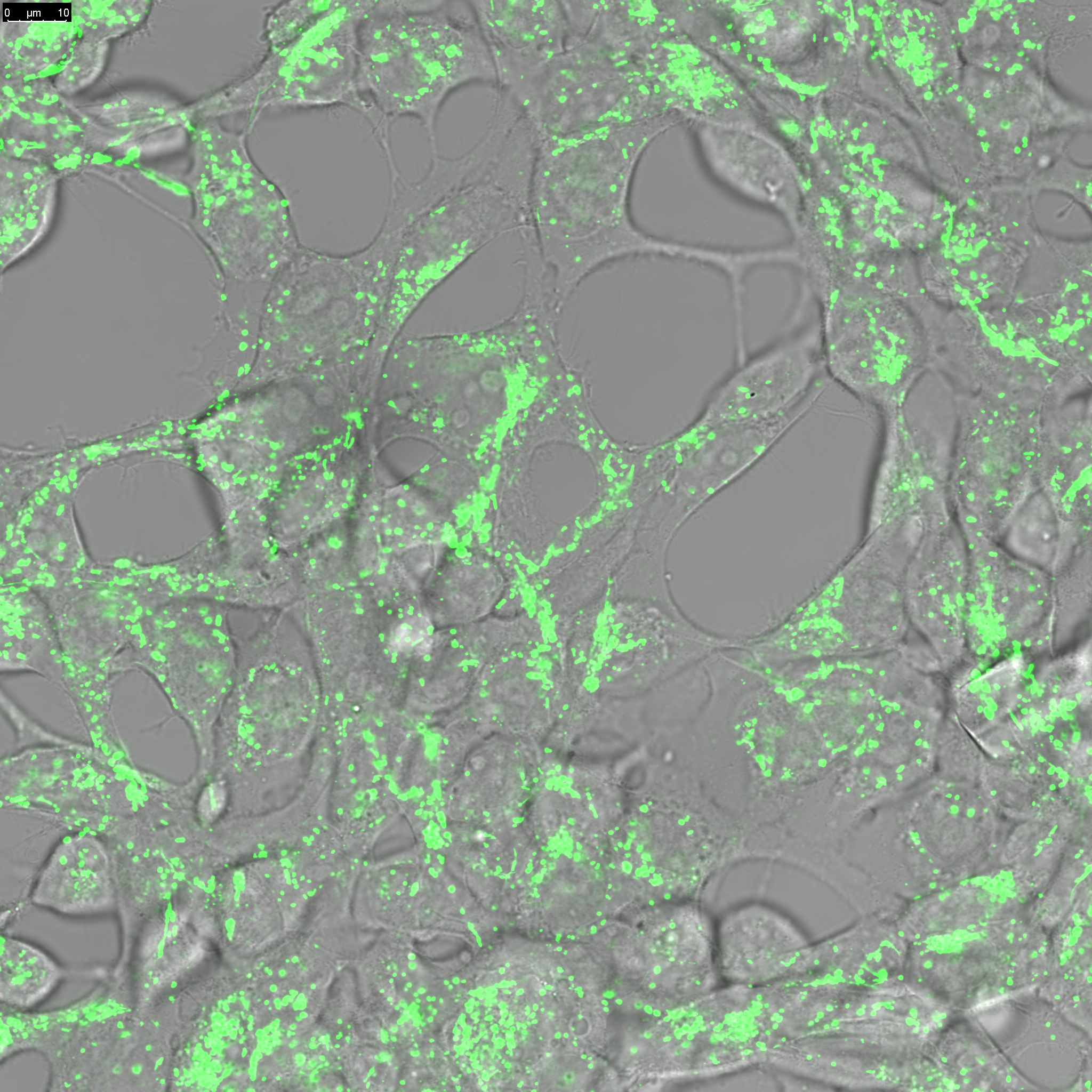

Supplement: Supplementary file 11 — Source data Fig. 5 [file 44319_2025_667_MOESM11_ESM.zip › Source_Data_Figure5/5A/Live_lysotrack_pCDNA_03.tif]

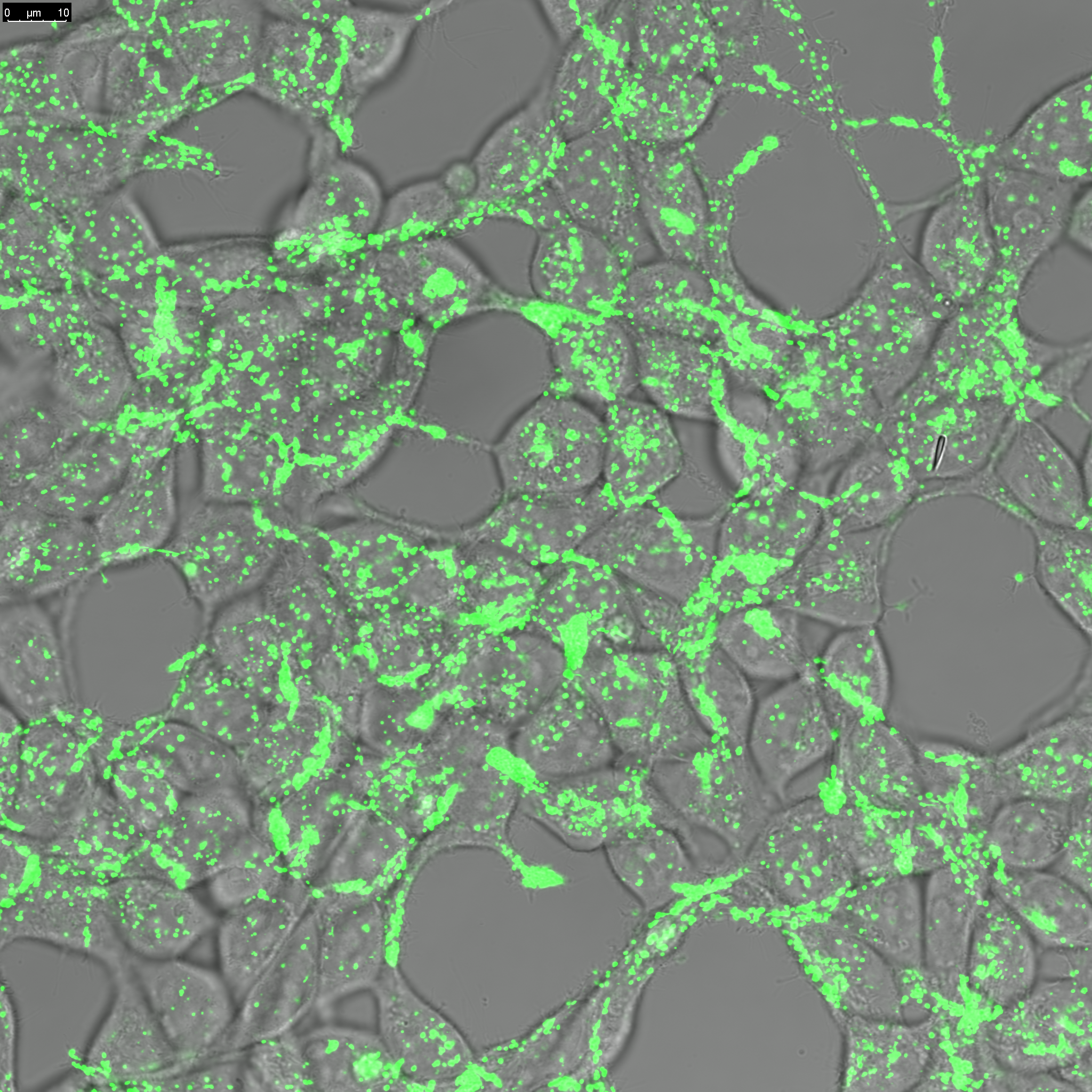

Supplement: Supplementary file 11 — Source data Fig. 5 [file 44319_2025_667_MOESM11_ESM.zip › Source_Data_Figure5/5A/Live_lysotrack_CtoS_05.tif]

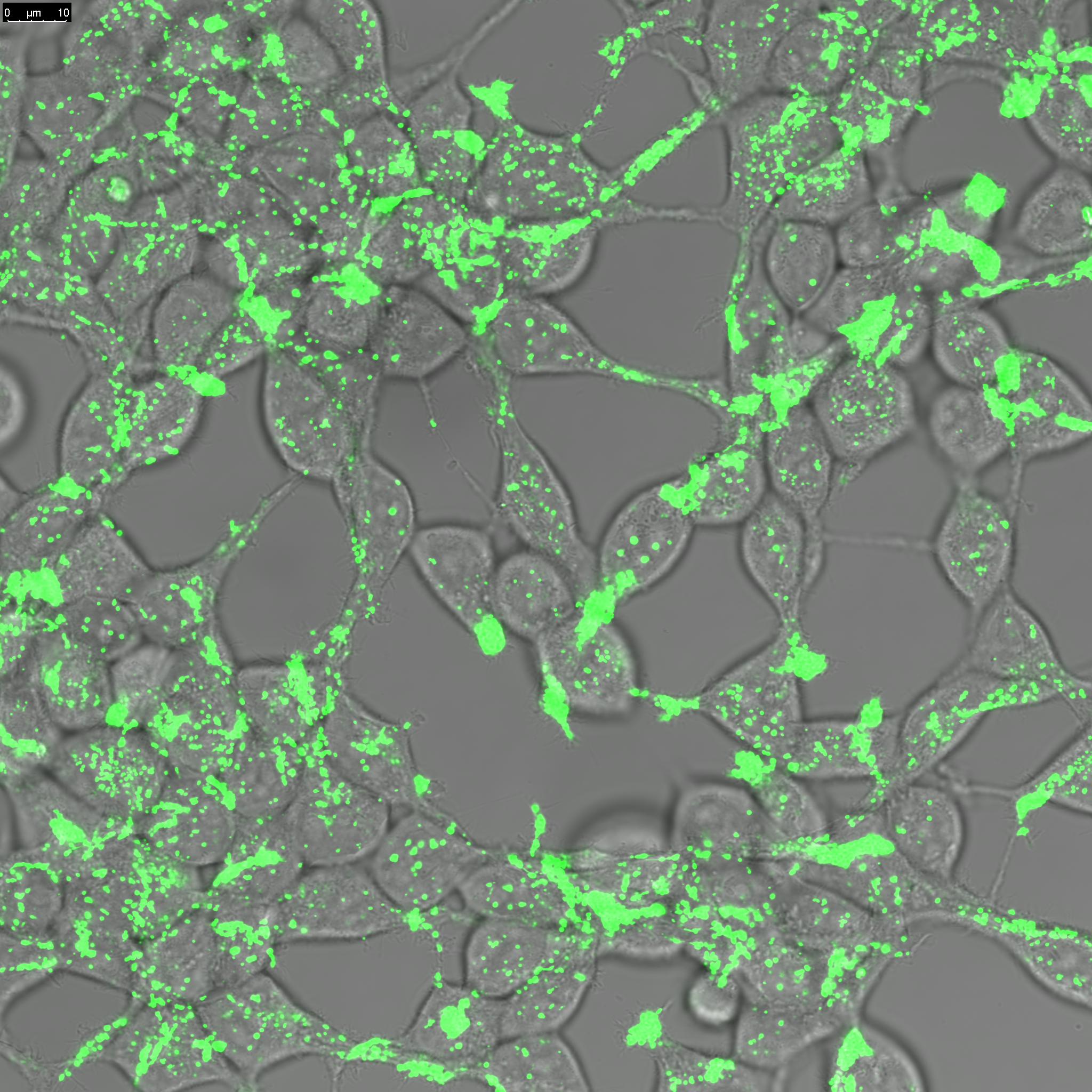

Supplement: Supplementary file 11 — Source data Fig. 5 [file 44319_2025_667_MOESM11_ESM.zip › Source_Data_Figure5/5A/Live_lysotrack_CtoS_04.tif]

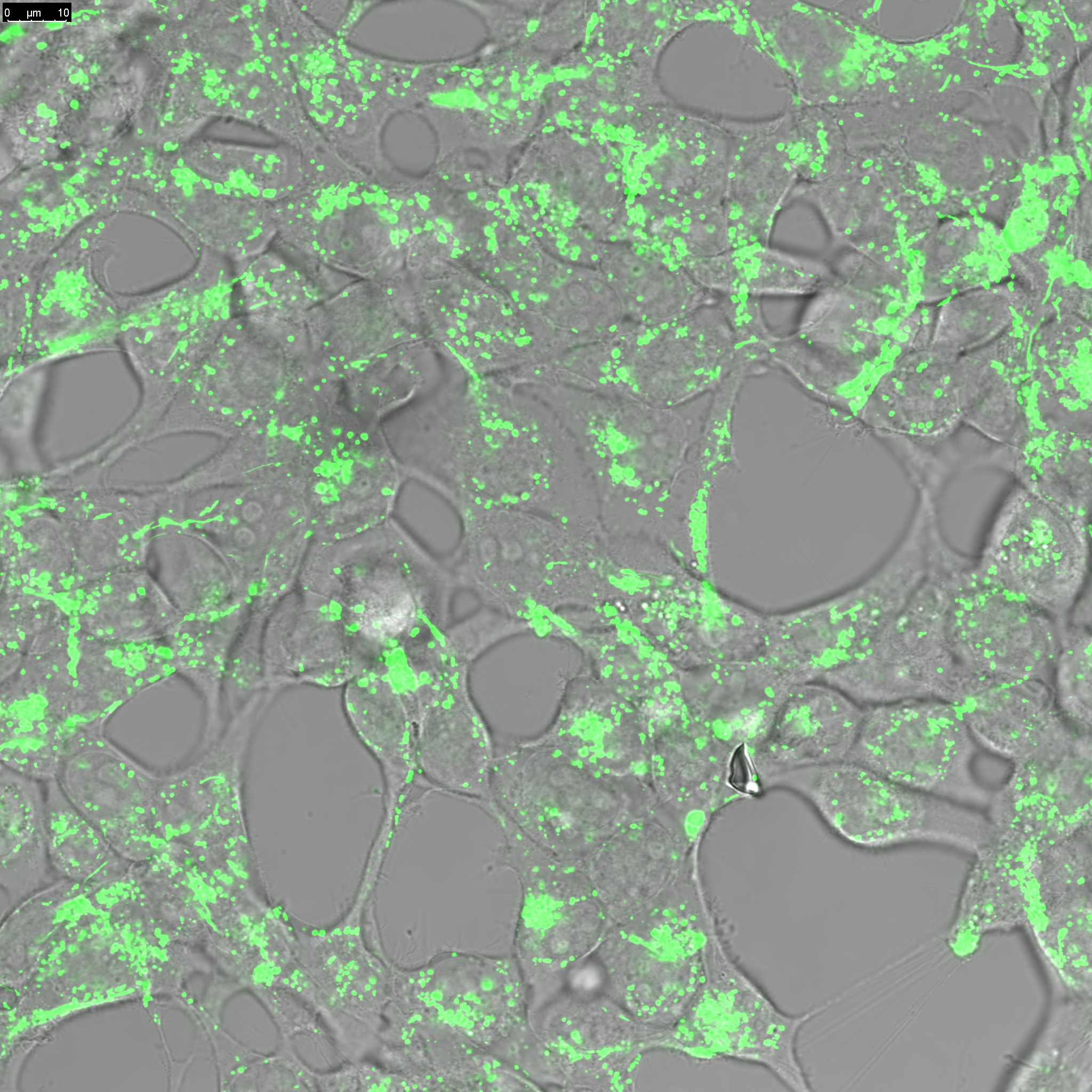

Supplement: Supplementary file 11 — Source data Fig. 5 [file 44319_2025_667_MOESM11_ESM.zip › Source_Data_Figure5/5A/Live_lysotrack_pCDNA_04.tif]

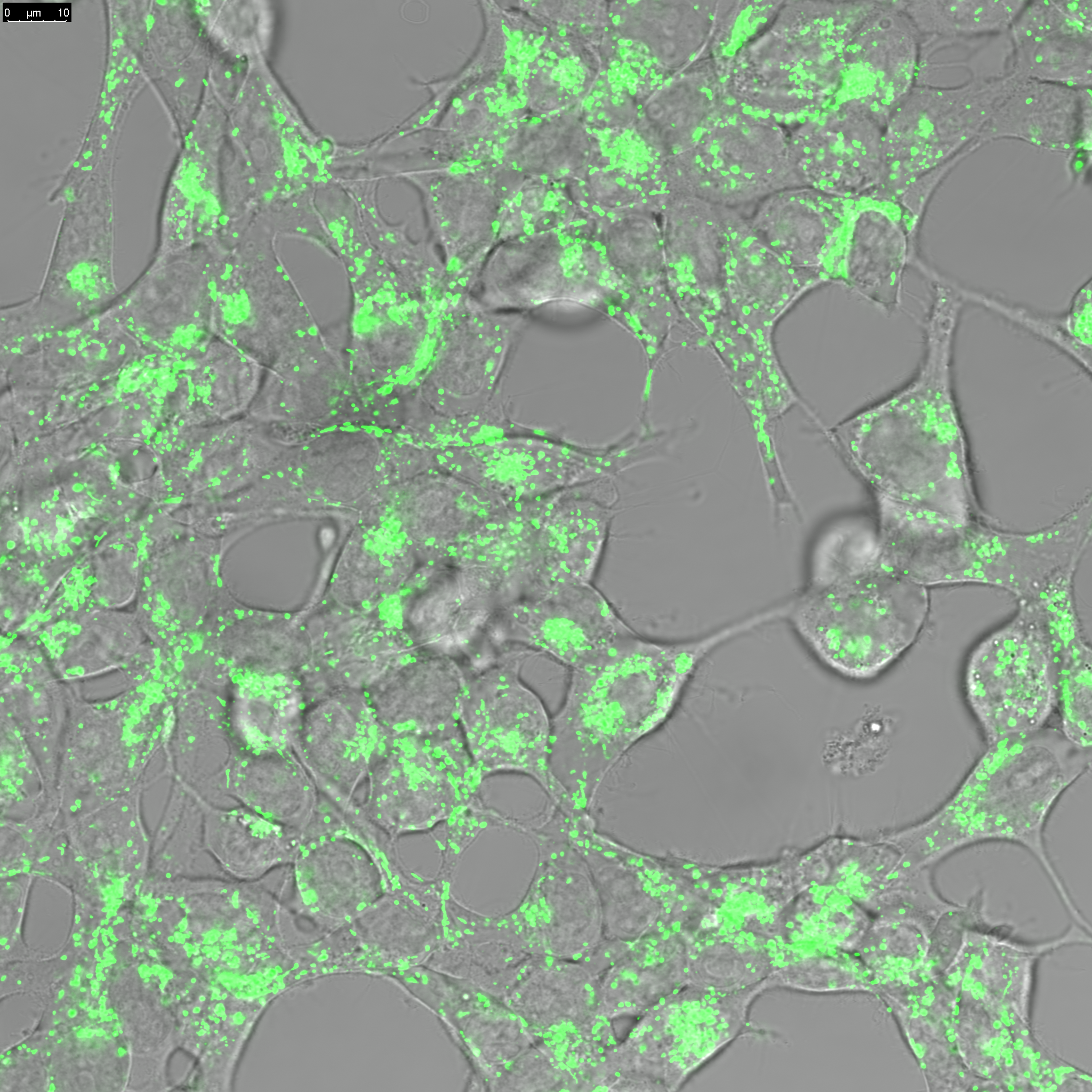

Supplement: Supplementary file 11 — Source data Fig. 5 [file 44319_2025_667_MOESM11_ESM.zip › Source_Data_Figure5/5A/Live_lysotrack_pCDNA_05.tif]

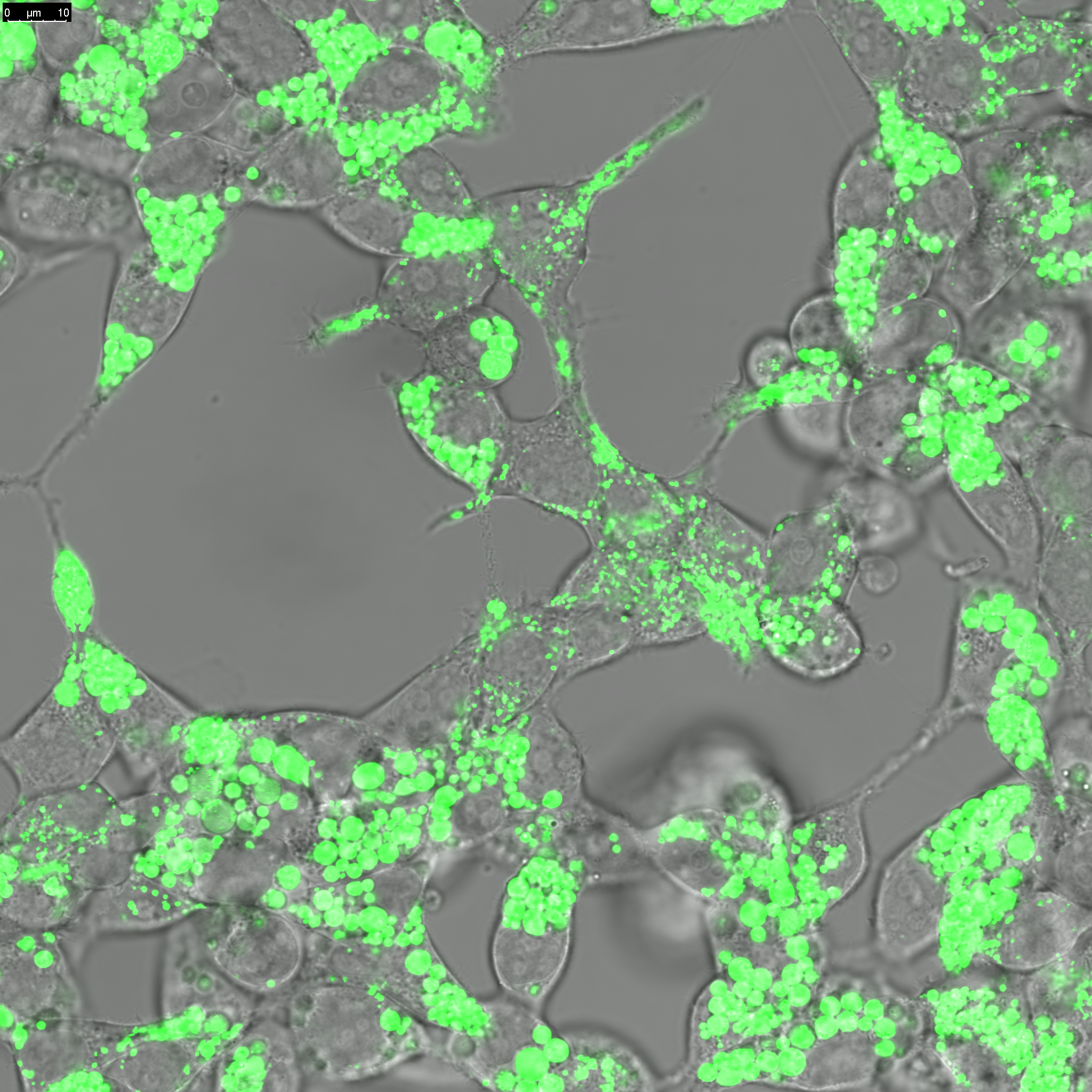

Supplement: Supplementary file 11 — Source data Fig. 5 [file 44319_2025_667_MOESM11_ESM.zip › Source_Data_Figure5/5A/Live_lysotrack_WT_01.tif]

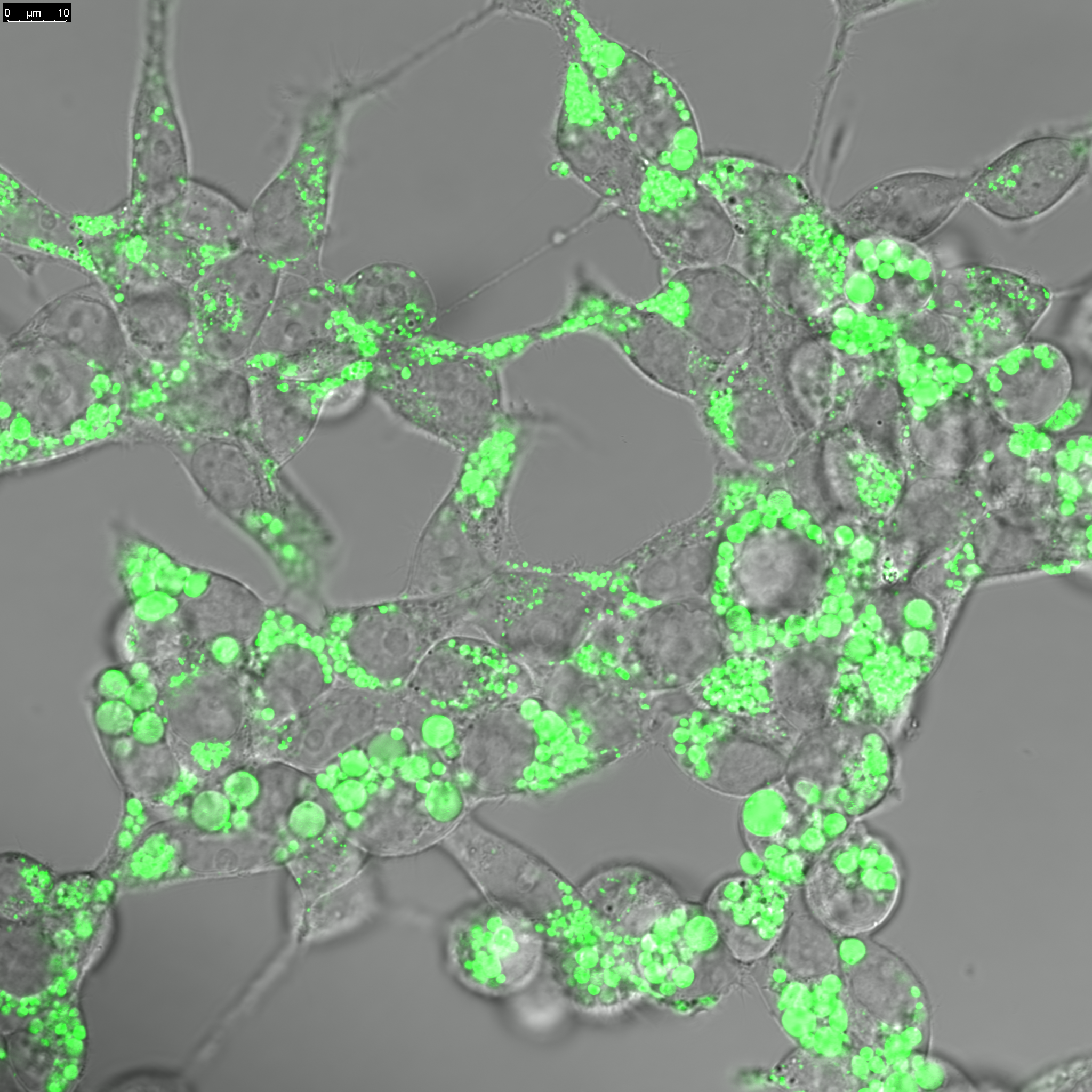

Supplement: Supplementary file 11 — Source data Fig. 5 [file 44319_2025_667_MOESM11_ESM.zip › Source_Data_Figure5/5A/Live_lysotrack_KtoA_05.tif]

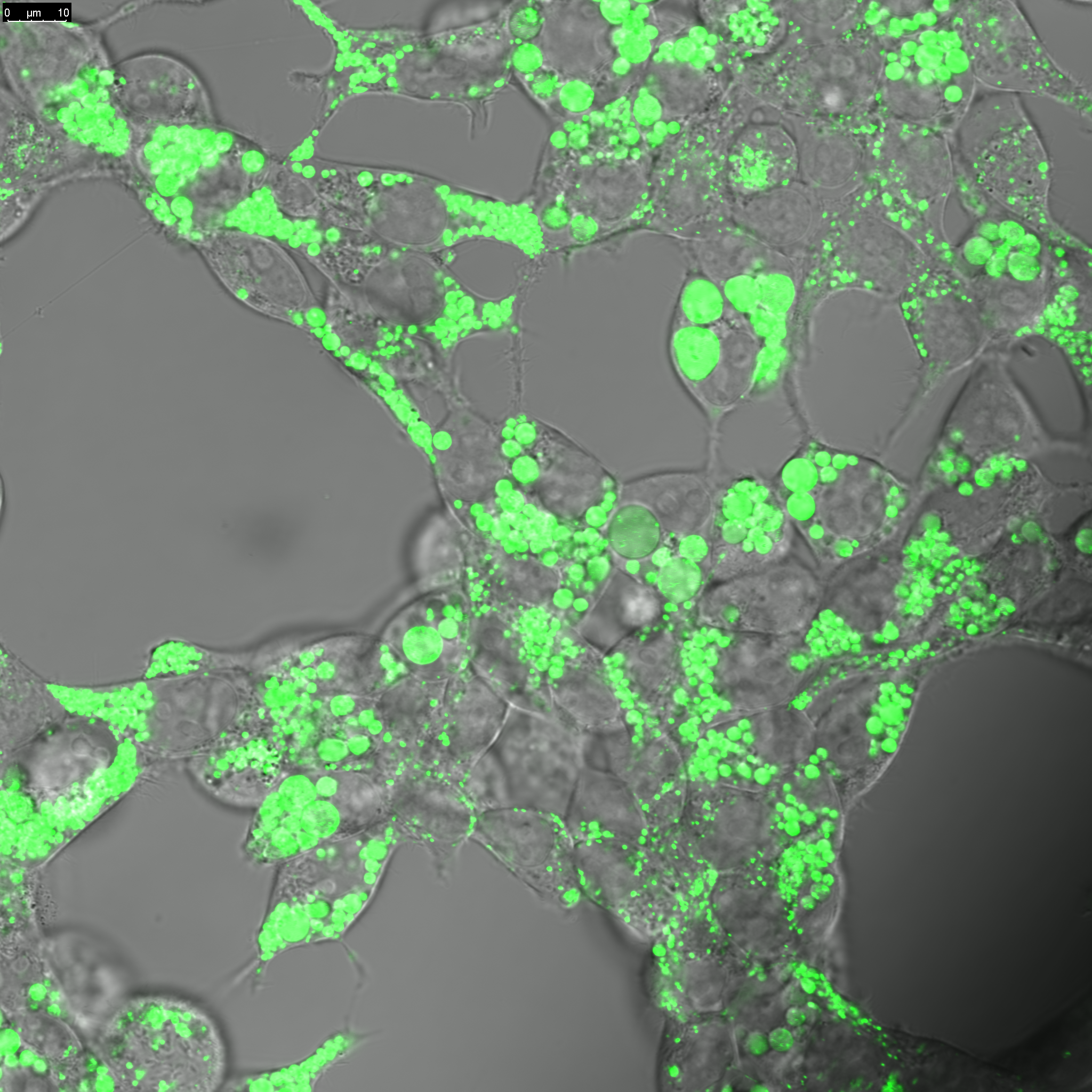

Supplement: Supplementary file 11 — Source data Fig. 5 [file 44319_2025_667_MOESM11_ESM.zip › Source_Data_Figure5/5A/Live_lysotrack_KtoA_04.tif]

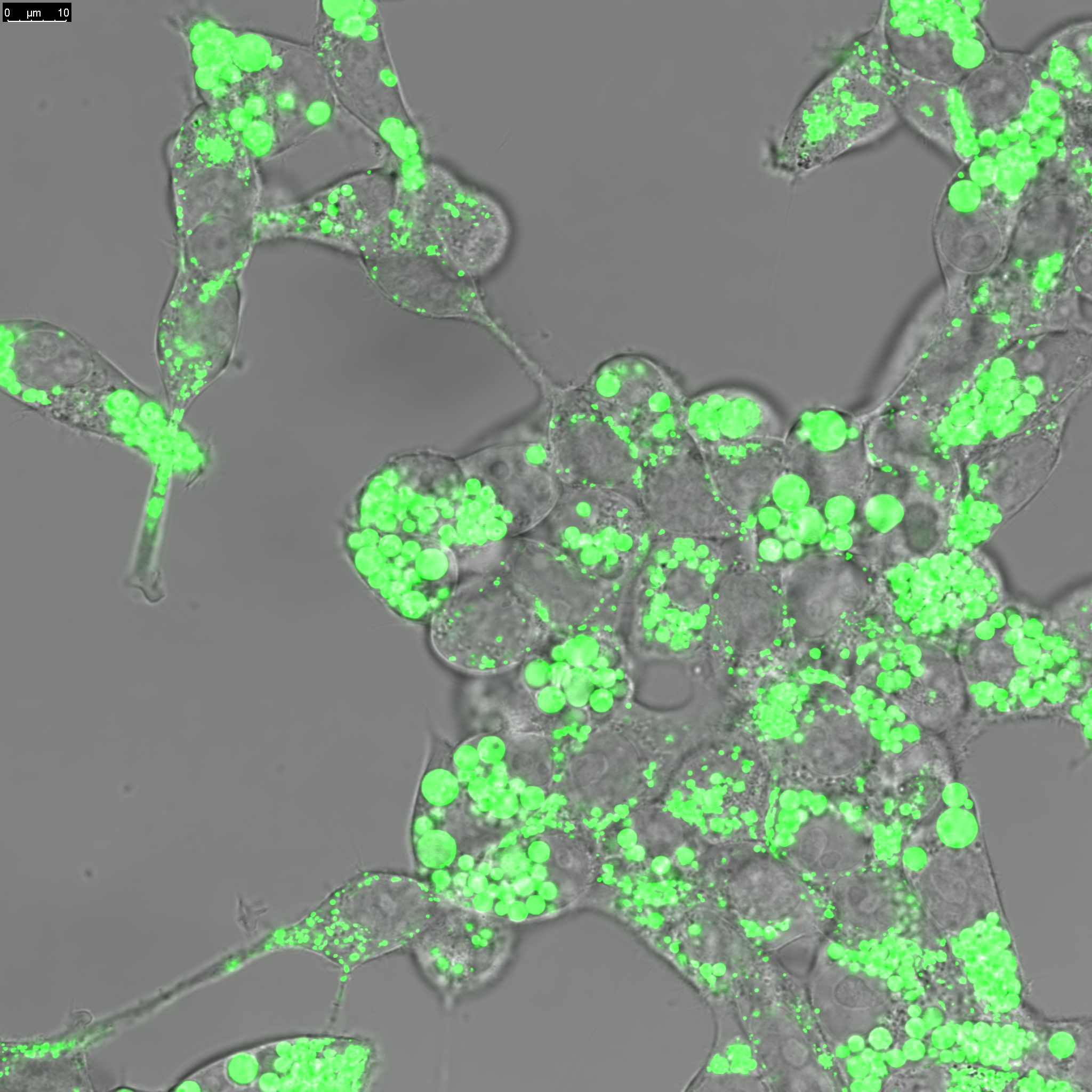

Supplement: Supplementary file 11 — Source data Fig. 5 [file 44319_2025_667_MOESM11_ESM.zip › Source_Data_Figure5/5A/Live_lysotrack_WT_02.tif]

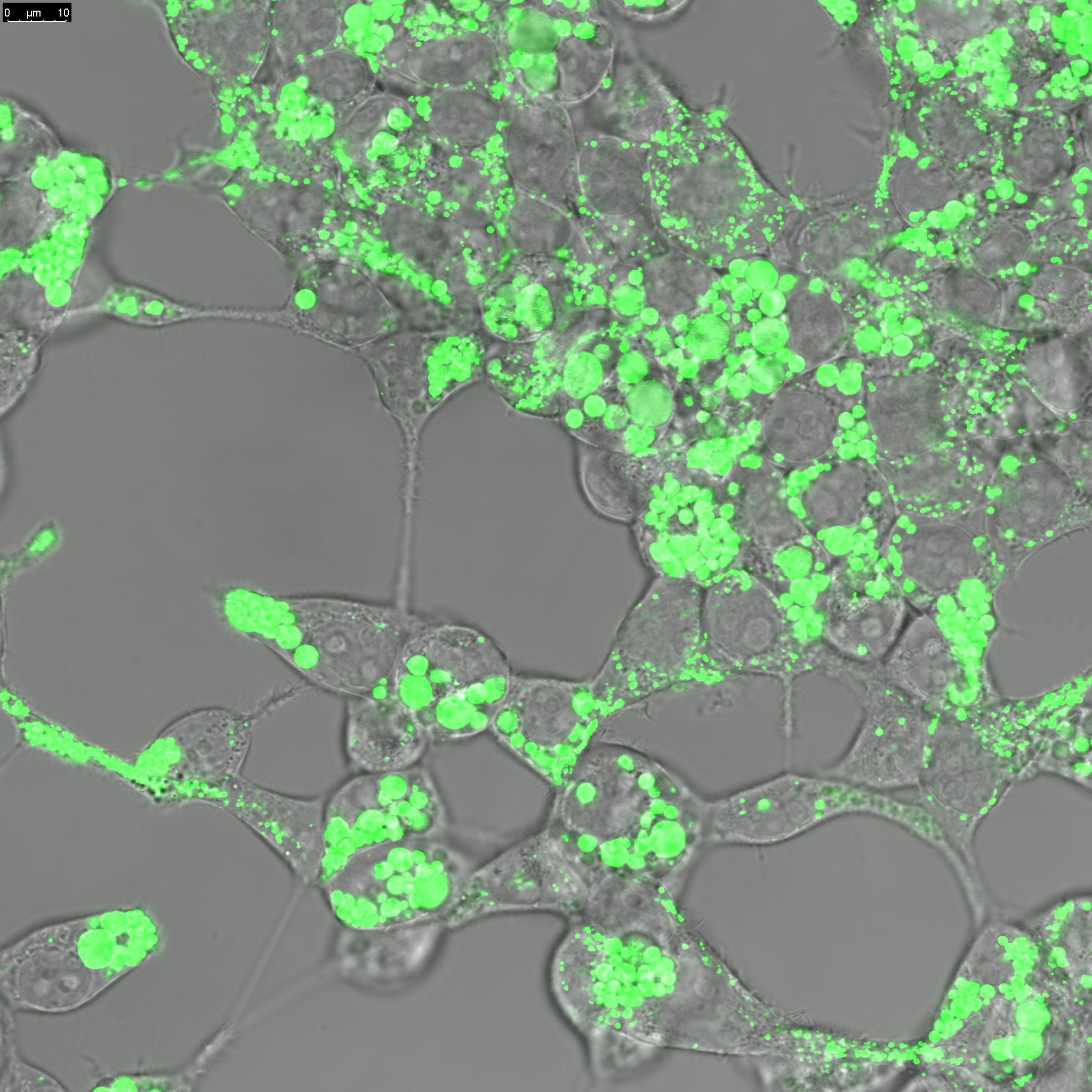

Supplement: Supplementary file 11 — Source data Fig. 5 [file 44319_2025_667_MOESM11_ESM.zip › Source_Data_Figure5/5A/Live_lysotrack_WT_03.tif]

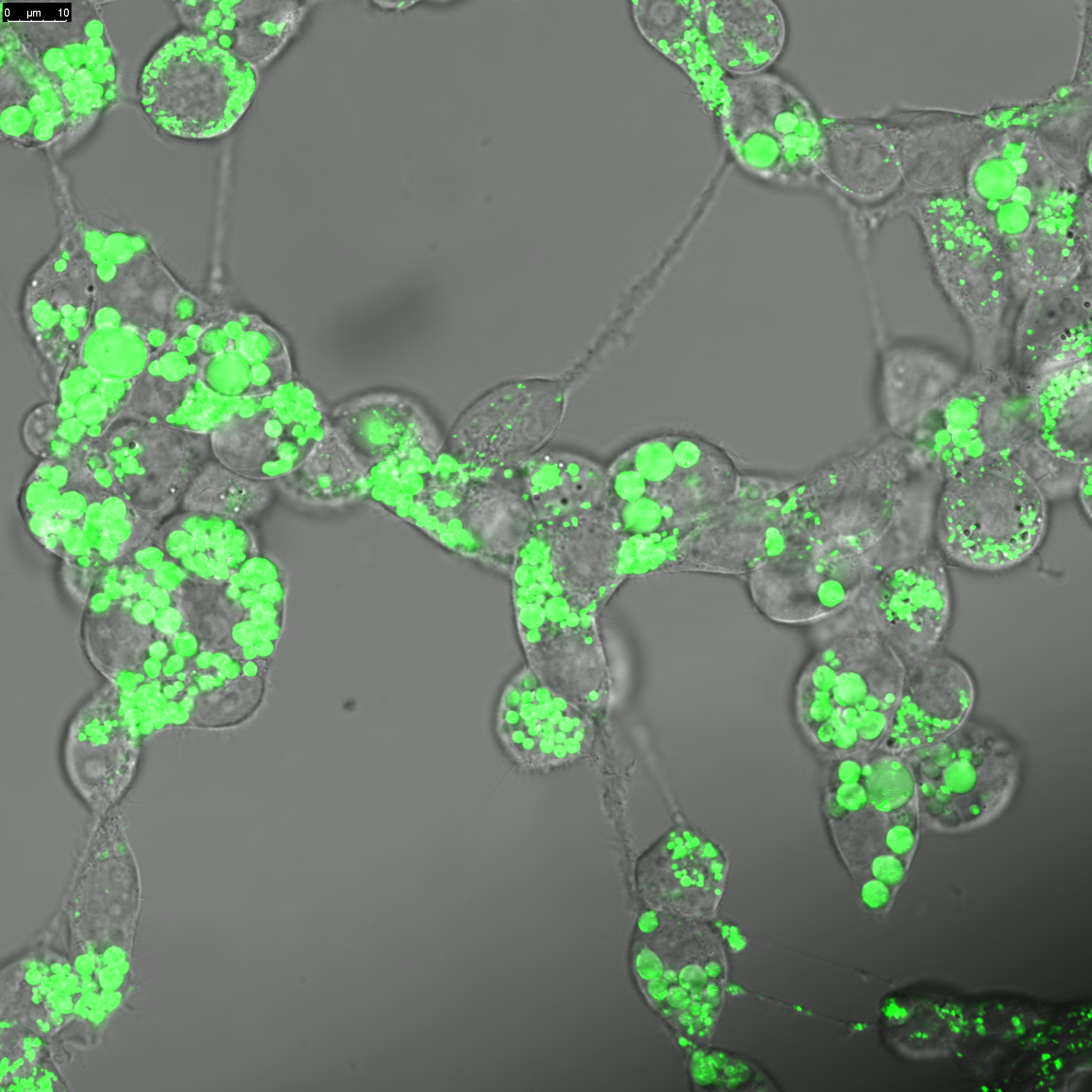

Supplement: Supplementary file 11 — Source data Fig. 5 [file 44319_2025_667_MOESM11_ESM.zip › Source_Data_Figure5/5A/Live_lysotrack_KtoA_03.tif]

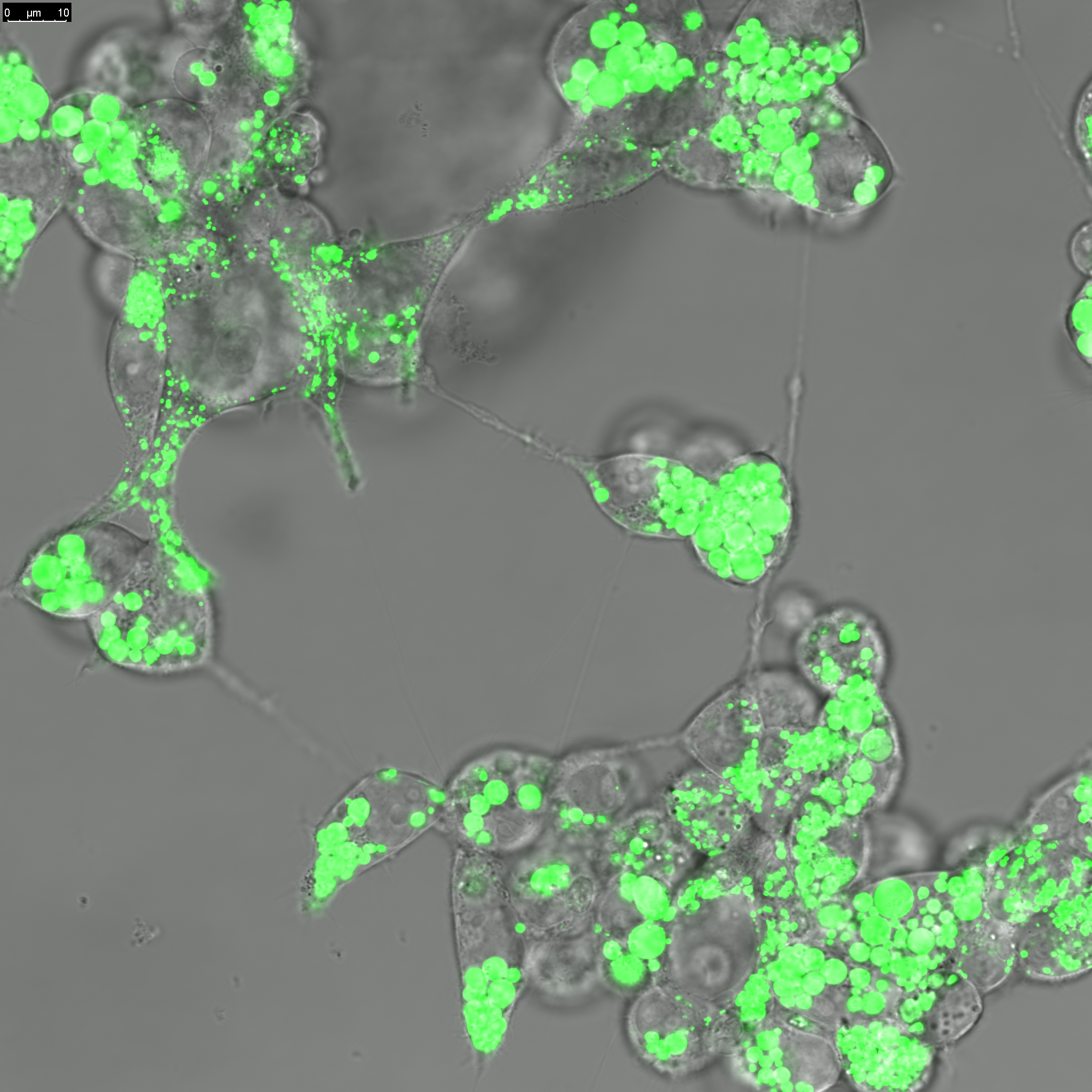

Supplement: Supplementary file 11 — Source data Fig. 5 [file 44319_2025_667_MOESM11_ESM.zip › Source_Data_Figure5/5A/Live_lysotrack_KtoA_02.tif]

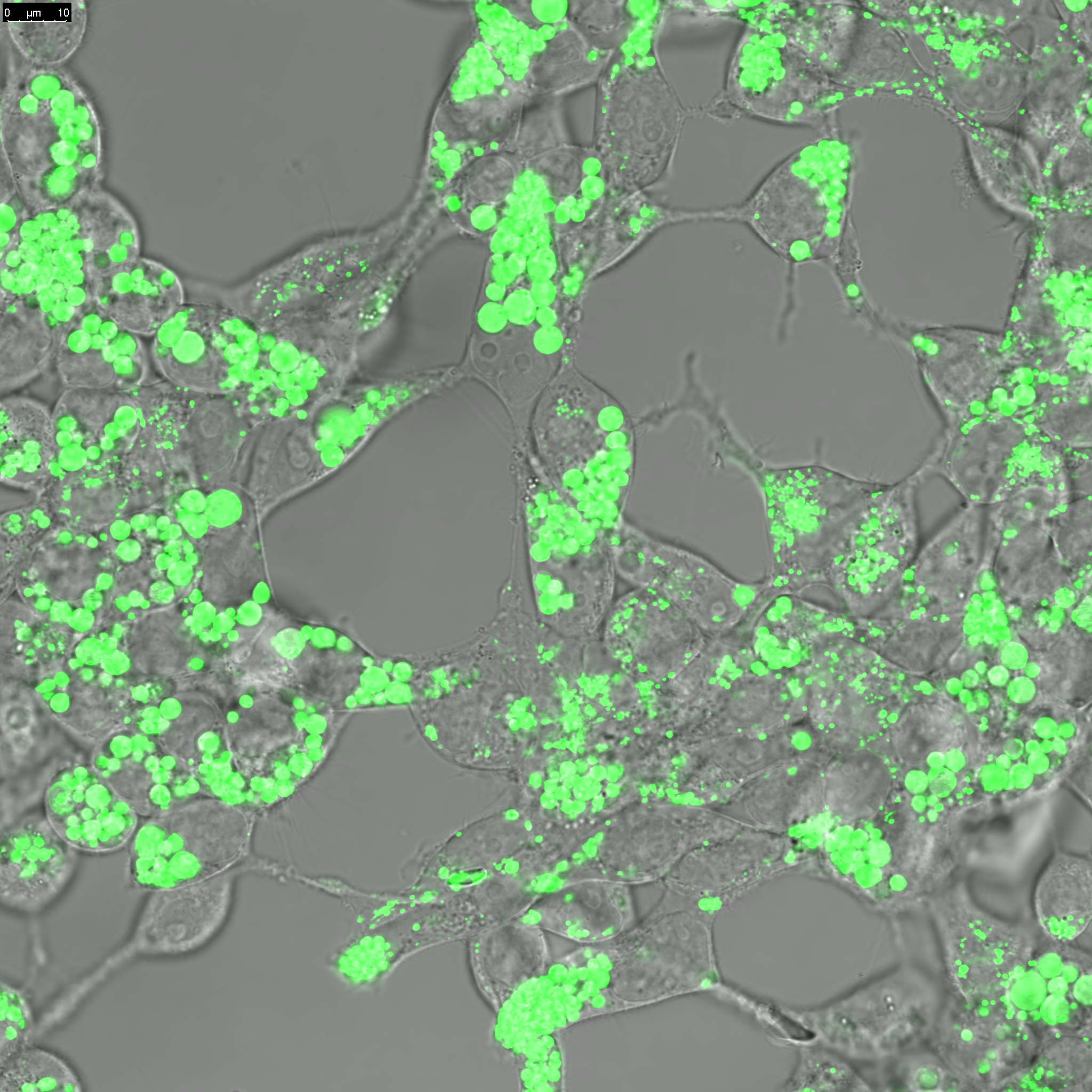

Supplement: Supplementary file 11 — Source data Fig. 5 [file 44319_2025_667_MOESM11_ESM.zip › Source_Data_Figure5/5A/Live_lysotrack_WT_04.tif]

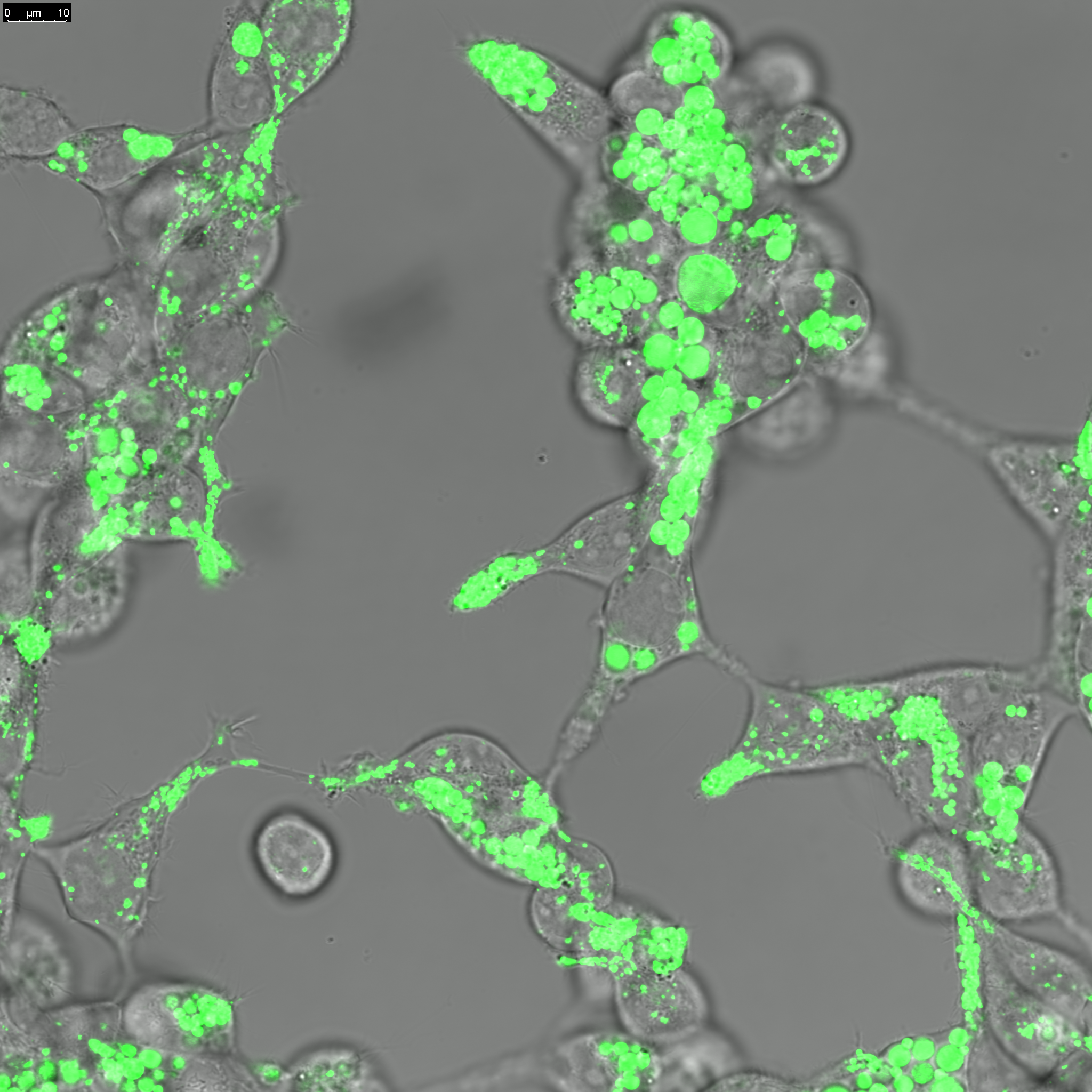

Supplement: Supplementary file 11 — Source data Fig. 5 [file 44319_2025_667_MOESM11_ESM.zip › Source_Data_Figure5/5A/Live_lysotrack_KtoA_01.tif]

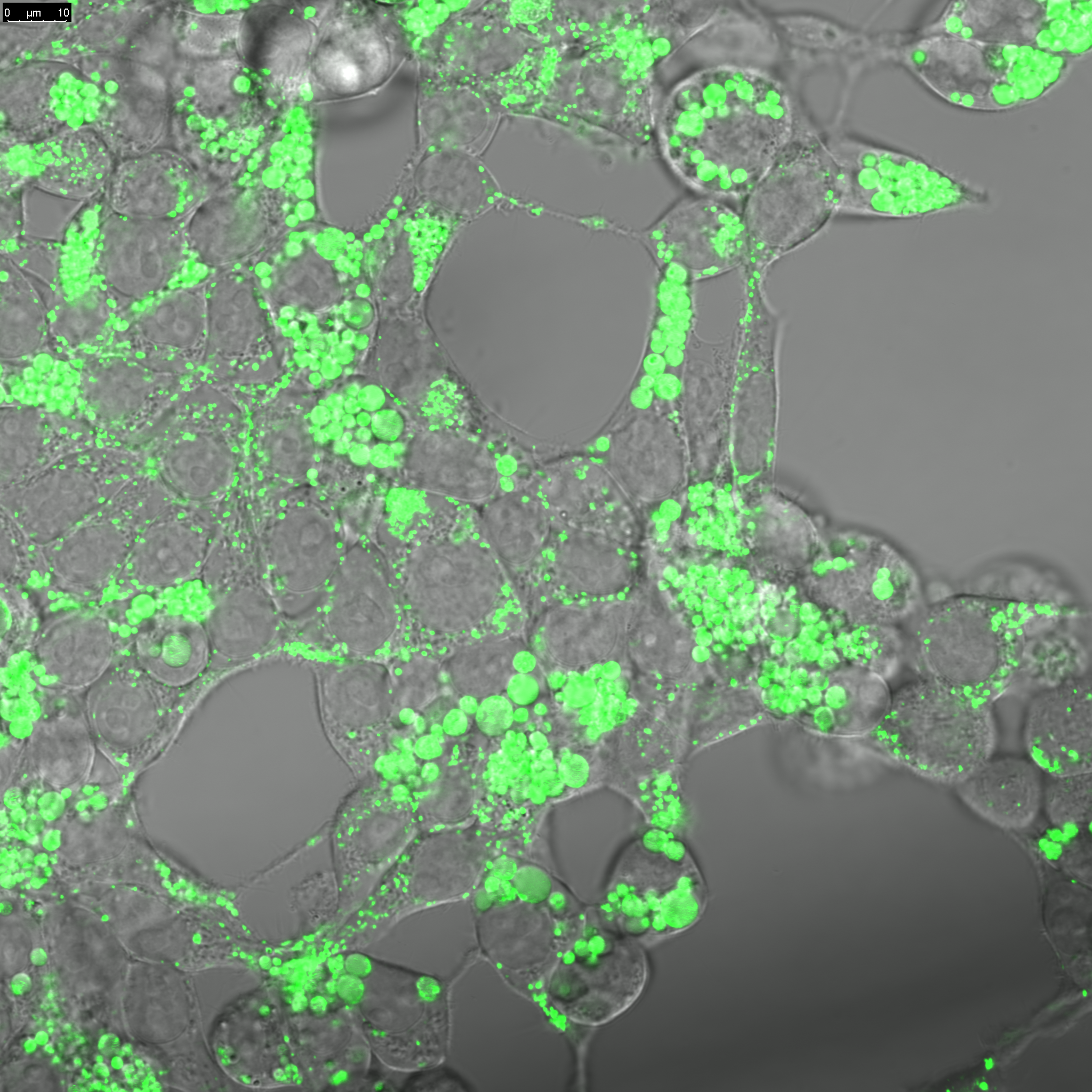

Supplement: Supplementary file 11 — Source data Fig. 5 [file 44319_2025_667_MOESM11_ESM.zip › Source_Data_Figure5/5A/Live_lysotrack_WT_05.tif]
