# Supplementary material for: TRIM2 E3 ligase substrate discovery reveals zinc-mediated regulation of TMEM106B in the endolysosomal pathway
Source: EMBO Rep. 2026 Jan 3;27(3):729–47. doi: 10.1038/s44319-025-00667-3 (PMC12894719; doi:10.1038/s44319-025-00667-3)
Supplement: Supplementary file 8 — Source data Fig. 2 [file 44319_2025_667_MOESM8_ESM.zip › Source_Data_Figure2/README.rtf]

Figure 2A: Schematic diagram, source data not applicable.Figure 2B: Western blots and SDS-PAGE, source data provided. The approximate region used in the manuscript has been highlighted in red. Figure 2C: Western blots, source data provided. The approximate region used in the manuscript has been highlighted in red. Figure 2D: Diagram from analysis of quantitative MS data deposited in a repository (see data availability section).
