# Supplementary material for: TRIM2 E3 ligase substrate discovery reveals zinc-mediated regulation of TMEM106B in the endolysosomal pathway
Source: EMBO Rep. 2026 Jan 3;27(3):729–47. doi: 10.1038/s44319-025-00667-3 (PMC12894719; doi:10.1038/s44319-025-00667-3)
Supplement: Supplementary file 9 — Source data Fig. 3 [file 44319_2025_667_MOESM9_ESM.zip › Source_Data_Figure3/README.rtf]

Figure 3A: 1H/15N-HSQC overlay of two spectra, source data provided as UCSF files (Sparky readable format) and as an Excel file. 
Figure 3B: Quantification of peak height changes (signal loss) derived from 1H/15N-HSQC spectra, provided as UCSF files (Sparky readable format) and as an Excel file.
Figure 3C: SEC-MALS, source data provided in an Excel file. 
Figure 3D: AlphaFold-Multimer relaxed output models for dimeric human TMEM106B 1-95, provided in PDB format. 
Figure 3E: 1H/15N-HSQC overlay of four spectra, source data provided as UCSF files (Sparky readable format). 
Figure 3F: 1H/15N-HSQC overlay of three spectra, source data provided as UCSF files (Sparky readable format). 
Figure 3G: The NMR structure has been deposited at the PBD (ID: 9GI8). 
