# Supplementary material for: TRIM2 E3 ligase substrate discovery reveals zinc-mediated regulation of TMEM106B in the endolysosomal pathway
Source: EMBO Rep. 2026 Jan 3;27(3):729–47. doi: 10.1038/s44319-025-00667-3 (PMC12894719; doi:10.1038/s44319-025-00667-3)
Supplement: Supplementary file 10 — Source data Fig. 4 [file 44319_2025_667_MOESM10_ESM.zip › Source_Data_Figure4/README.rtf]

Figure 4A: 1H/15N-HSQC overlay of two spectra, source data provided as UCSF files (Sparky readable format). 
Figure 4B: Quantification of peak height changes (signal loss) derived from 1H/15N-HSQC spectra. Source data provided as an Excel file.
Figure 4C: Blots and SDS-PAGE, source data provided. 
