# Supplementary material for: TRIM2 E3 ligase substrate discovery reveals zinc-mediated regulation of TMEM106B in the endolysosomal pathway
Source: EMBO Rep. 2026 Jan 3;27(3):729–47. doi: 10.1038/s44319-025-00667-3 (PMC12894719; doi:10.1038/s44319-025-00667-3)
Supplement: Supplementary file 11 — Source data Fig. 5 [file 44319_2025_667_MOESM11_ESM.zip › Source_Data_Figure5/README.rtf]

Figure 5A: Images collected of HEK293-T cells transfected with TMEM106B FL variants and lysosomal size quantification. Source data provided in TIFF format (images) or in an Excel file (quantification). The lysosomal signal was arbitrarily assigned the green channel for better visualization but corresponds to LysoRed detection (see Methods). Each image contains two channels: the transmission and LysoRed signals (wavelength ~ 600 nm). 
Figure 5B: Volcano scatter plot from quantitative MS data deposited in a repository (see data availability section).
Figure 5C: Schematic of working model, source data not applicable. 
